# Anatomical variations of the deep head of Cruveilhier of the flexor pollicis brevis and its significance for the evolution of the precision grip

**DOI:** 10.1371/journal.pone.0187402

**Published:** 2017-11-09

**Authors:** Samuel S. Dunlap, M. Ashraf Aziz, Janine M. Ziermann

**Affiliations:** 1 Independent Researcher, Reston, VA, United States of America; 2 Howard University College of Medicine, Dept. Anatomy, Washington, DC, United States of America; University of Utah, UNITED STATES

## Abstract

Cruveilhier described in 1834 the human flexor pollicis brevis (FPB), a muscle of the thenar compartment, as having a superficial and a deep head, respectively, inserted onto the radial and ulnar sesamoids of the thumb. Since then, Cruveilhier’s deep head has been controversially discussed. Often this deep head is confused with Henle’s “interosseous palmaris volaris” or said to be a slip of the oblique adductor pollicis. In the 1960s, Day and Napier described anatomical variations of the insertions of Cruveilhier’s deep head, including its absence, and hypothesized, that the shift of the deep head’s insertion from ulnar to radial facilitated “true opposability” in anthropoids. Their general thesis for muscular arrangements underlying the power and precision grip is sound, but they did not delineate their deep head from Henle’s muscle or the adductor pollicis, and their description of the attachments of Cruveilhier’s deep head were too vague and not supported by a significant portion of the anatomical literature. Here, we reinvestigated Cruveilhier’s deep head to resolve the controversy about it and because many newer anatomy textbooks do not describe this muscle, while it is often an obvious functionally (writing, texting, precision grip) and clinically significant thenar muscle. For the first time, we empirically delineated Cruveilhier’s deep head from neighboring muscles with which it was previously confused. We observed 100% occurrence of the uncontested deep head in 80 human hands, displaying a similar variability of insertions as Day and Napier, but in significantly different numbers. Furthermore, we found variability in the origin and included as important landmarks the trapezoid and the ligamentum carpi radiatum. We tested the assertion regarding the evolutionary morphology and its role in the improvements in thumb movements during various precision grips. Our overall conclusions differ with respect to the developmental and evolutionary origin of the FPB heads.

## Introduction

**Fig 1:**
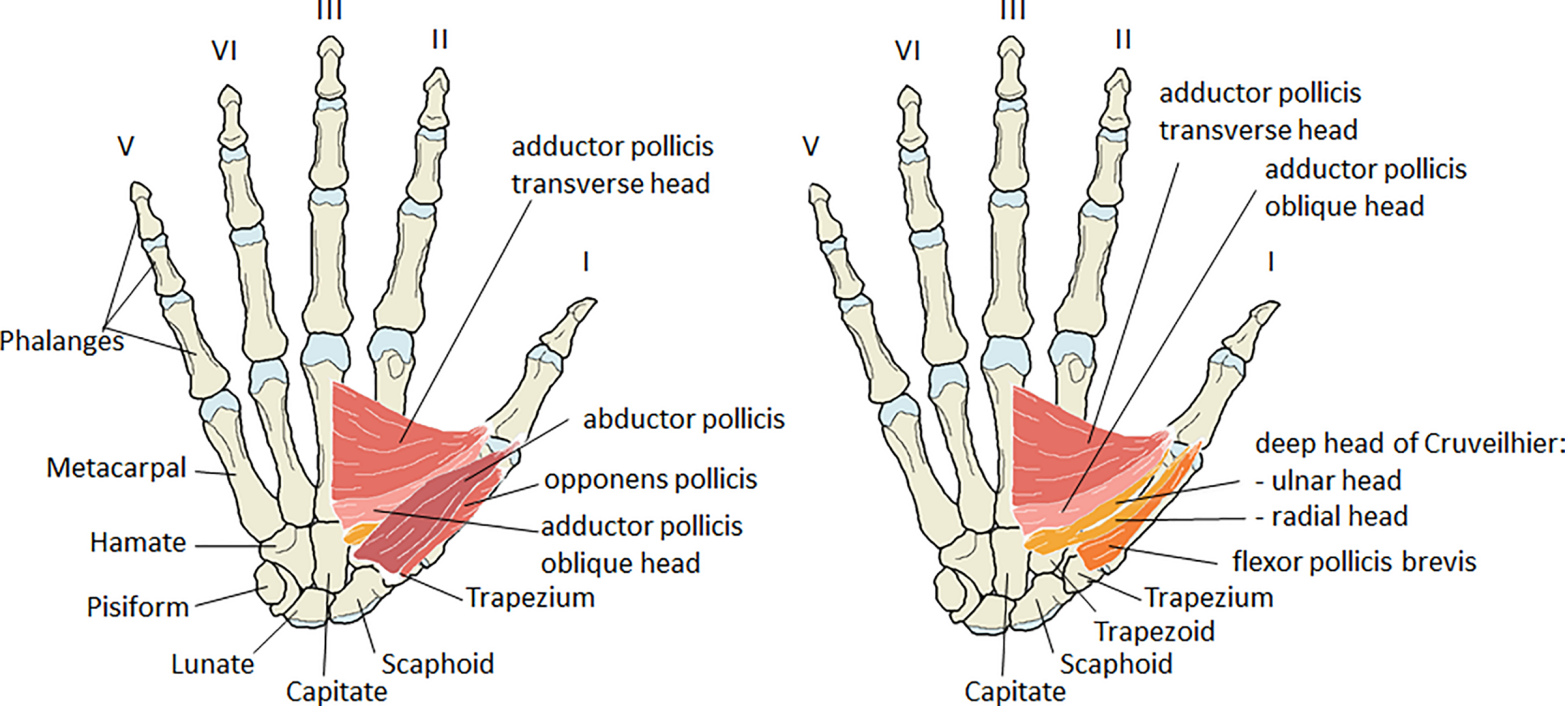
Schematic drawings of the right hand, palmar view, with the thenar muscles shown in two layers. Only bony attachments are shown. **Left)** Overview of the thenar muscles as visible after skinning the hand. Both heads of the flexor pollicis brevis are covered by the abductor pollicis. **Right)** Abductor pollicis and opponens pollicis are completely removed. The deep head of Cruveilhier (orange; a.k.a. the deep head of the flexor pollicis brevis) is shown with two heads attaching onto the ulnar and radial side at the base of the proximal phalanx of the thumb. The superficial head of the flexor pollicis brevis (dark orange) has a single head that inserts ulnarward onto the proximal phalanx of the thumb.

The muscles in the human thumb, especially those in the thenar compartment (Fig 1), confer on the *Homo sapiens* hand a unique combination of a powerful grip and delicate manipulative abilities particularly between the first two digits. These movements occur around the saddle-shaped, trapezio-metacarpal joint and include others, i.e., carpo-metacarpal, metacarpo-phalangeal, and interphalangeal joints: all activated by the largest number of extrinsic and intrinsic pollical muscles of any primate [1–13]. However, this should not be confused with the total number of hand muscles as apes and primates have equal or often more muscles in their hands [14].

The precise actions of the pollical muscles are well understood; however, considerable variations exist in the morphology and appearance of most of these muscles [15–18]. The deep head of the flexor pollicis brevis (FPB)–often named the deep head of Cruveilhier–has probably been the most misunderstood of all the muscles in the thumb or even hand (for a review on the history of the muscle of Cruveilhier see [19]). Ostensibly, its appearance in the anatomical literature commences with Albinus’s [20] description of a two headed FPB. Subsequently, Henle [21] described a first palmar interosseous (interosseous palmaris volaris = IPV, interosseous volaris primus, interosseous palmaris I of Henle, or Henle’s muscle) while accepting the two heads of FPB. Ensuing studies of these closely lying muscles and the adjacent oblique head of adductor pollicis illustrate considerable confusion concerning the arrangement, morphology, innervation, and even existence of some of these muscles [22–27].

The confusion was not cleared up by attempts at a phylogenetic approach [28–33]. In his book on the human hand Wood Jones [15] stressed the variability of these muscles and he attempted to resolve issues by clearly describing origins, insertions, and morphology of all the muscles. Finally, Day and Napier [34] undertook a systematic dissection of 65 Human hands followed in 1963 by the dissection of 27 different primate genera (41 hands [35]). Before and during the intervening years several studies on the thumb muscles clearly established the constant incidence of the interosseous palmaris I of Henle [36–40]. Additionally, phylogenetic and ontogenetic investigations have continued to advance our knowledge of the human thumb [12–14, 41–46].

Moreover, specifically regarding the deep head of Cruveilhier, we have followed up on these studies on the human hand because confusion has continued about the morphology, incidence, innervation, ontogeny and phylogeny of this muscle. We are reinvestigating the deep head of Cruveilhier because although Day and Napier’s [34] general thesis for the muscular arrangements underlying the power and precision grip is sound, their description of the origins and insertions of the FPB deep head were too vague and not supported by a significant portion of the anatomical literature. Additionally, the deep head’s relationship with the closely associated interosseous palmaris I of Henle and the oblique head of the adductor pollicis was not clearly demonstrated.

Another important reason to reinvestigate the FPB and its slips is, that many newer anatomy atlases and textbooks do not show or describe the deep head of Cruveilhier, while it is often a very obvious functionally and clinically significant muscle of the thenar compartment (Fig 1). Medical students in particular should be aware of this muscle and its contribution to the subtle movements of the thumb. Besides, the use of enhanced fine surgical techniques now facilitates the re-attachments of severed digits or even wrist and hand [47, 48]; these surgeries require accurate anatomical information. We therefore: 1) analyze the frequency of the presence of the muscle of Cruveilhier and its positional and functional relationship to the “muscle of Henle” and to the oblique head of the adductor pollicis; 2) describe the origins, insertions, innervations, and the variable form of this muscle; and 3) discuss the results in the light of the evolution of the precision grip, medical importance, and evolution. The presented study contributes to the accurate understandings and descriptions of thenar muscles what will be useful in education, and can contribute to better surgical outcomes and more accurate phylogenetic analyses.

## Material and methods

We have completed thenar compartment dissections on 80 adult human hands (31 paired, 18 isolated hands: ten isolated lefts; eight isolated rights; all presumably karyotypically normal; S1 Table) obtained from the Anatomy Department’s “The Anatomy Donor Program, Howard University, College of Medicine, Department of Anatomy” (Washington, DC) and “Maryland Anatomy Board's Body Donation Program” at the University of Maryland College of Medicine (Baltimore, MD). Very few superficial dissections had been performed by medical, dental, and/or physical therapy students and these cases were available for deep dissection. We selected only those hands with undisturbed thenar musculature and only superficial investigations of the forearm. Our dissections were focused on the investigation of the deep thenar muscle morphology. We also traced the innervation of these muscles in 11 hands upon which no previous dissection had been conducted. The dissection sequence generally followed instructions by Romanes [49].

Dissections were carried out using gross dissection and micro-dissection tools and a three-power lighted lens and, as needed, a binocular dissecting microscope (Nikon SMZ1500). Terminology follows Dunlap and Aziz [12, 44] with the exception of the oblique head of the adductor pollicis (contrahens I in [12, 44]). Dissections were recorded by hand drawings, annotations, and photographs (Nikon digital camera D90). Because our dissections are meant to compare with the important work of Day and Napier [34, 35] our sample has been subjected to the appropriate statistical test (Chi square goodness of fit). Quantifiable data are tabulated in S1 Table indicating sex, origin, insertion, and the presence of the muscle of Henle; various exemplary dissections are illustrated in our figures.

## Results

**Fig 2:**
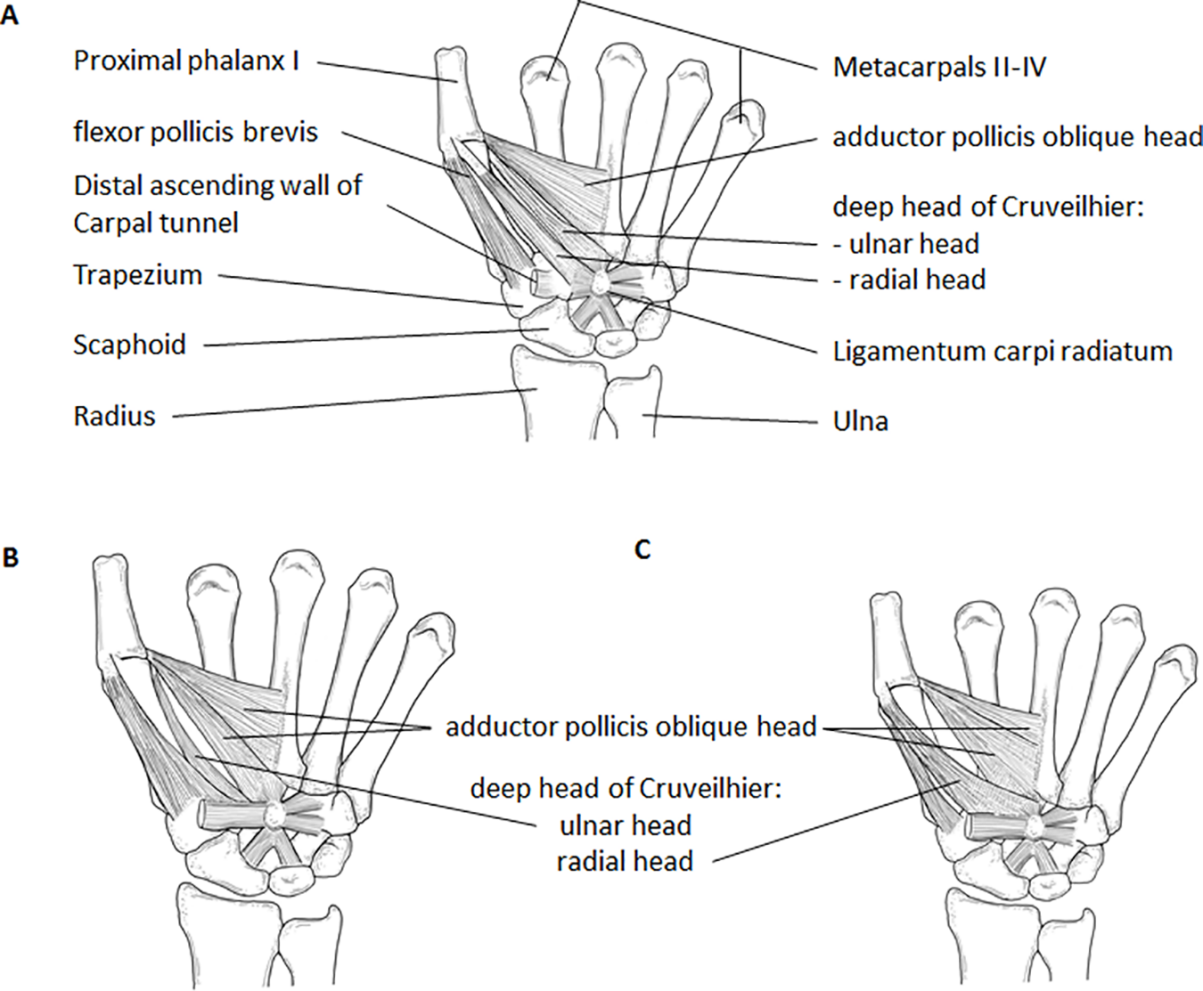
Schematic drawings of three possible configurations for the deep head of Cruveilhier. **A)** Overview of muscles attaching onto the proximal phalanx of the thumb. The deep head of Cruveilhier has two heads that insert radialward and ulnarward onto the base of the proximal phalanx 1. **B)** The deep head of Cruveilhier with only ulnar insertion. In some specimens, like the one depicted here, the most radialward fibers of the oblique head of the adductor pollicis form another slip that can be mistaken for the deep head of Cruveilhier. However, the origin is continuous with the fibers of the rest of the oblique adductor pollicis. **C)** The deep head of Cruveilhier with only radial insertion. Drawings by Marie Dauenheimer.

**Fig 3:**
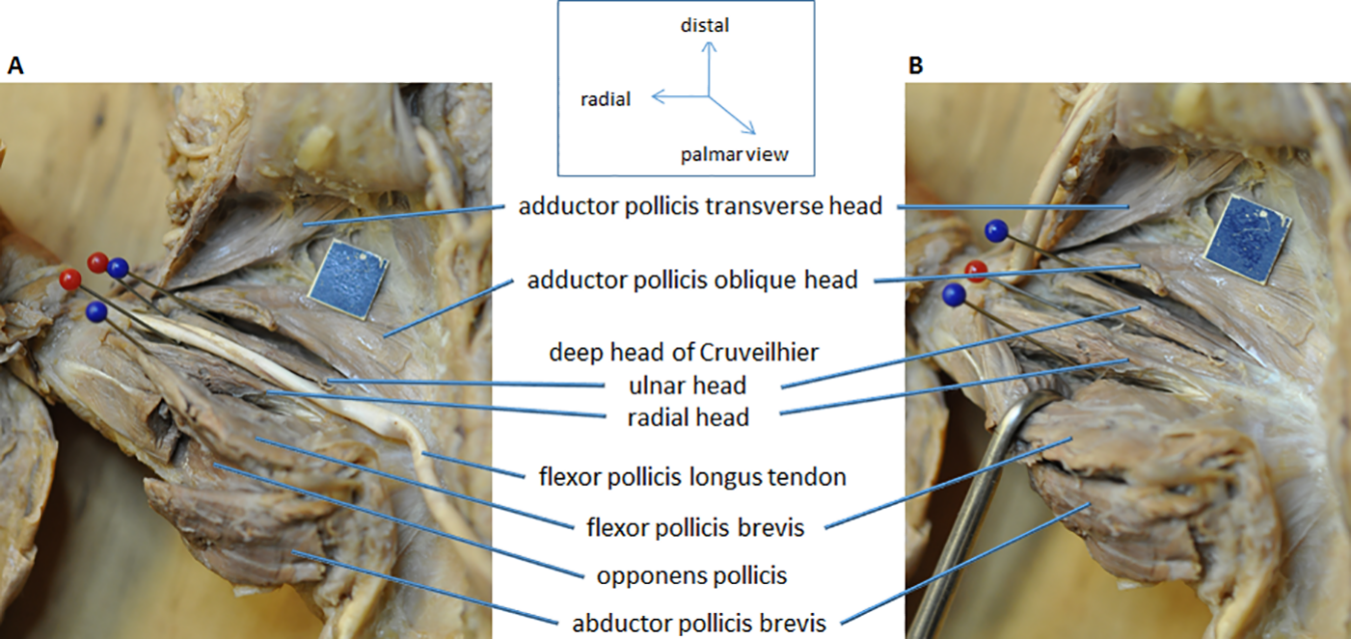
Palmar view of left hand (#754) with split deep head of Cruveilhier. Split head of Cruveilhier inserting onto the ulnar and radial sesamoid at the proximal phalanx of the thumb. **A)** Abductor pollicis reflected to show the opponens pollicis. **B)** Tendon of the flexor pollicis longus reflected and superficial head of the flexor pollicis brevis retracted for better view of the origin and insertion of the deep head of Cruveilhier. Blue scale = 1 cm.

**Fig 4:**
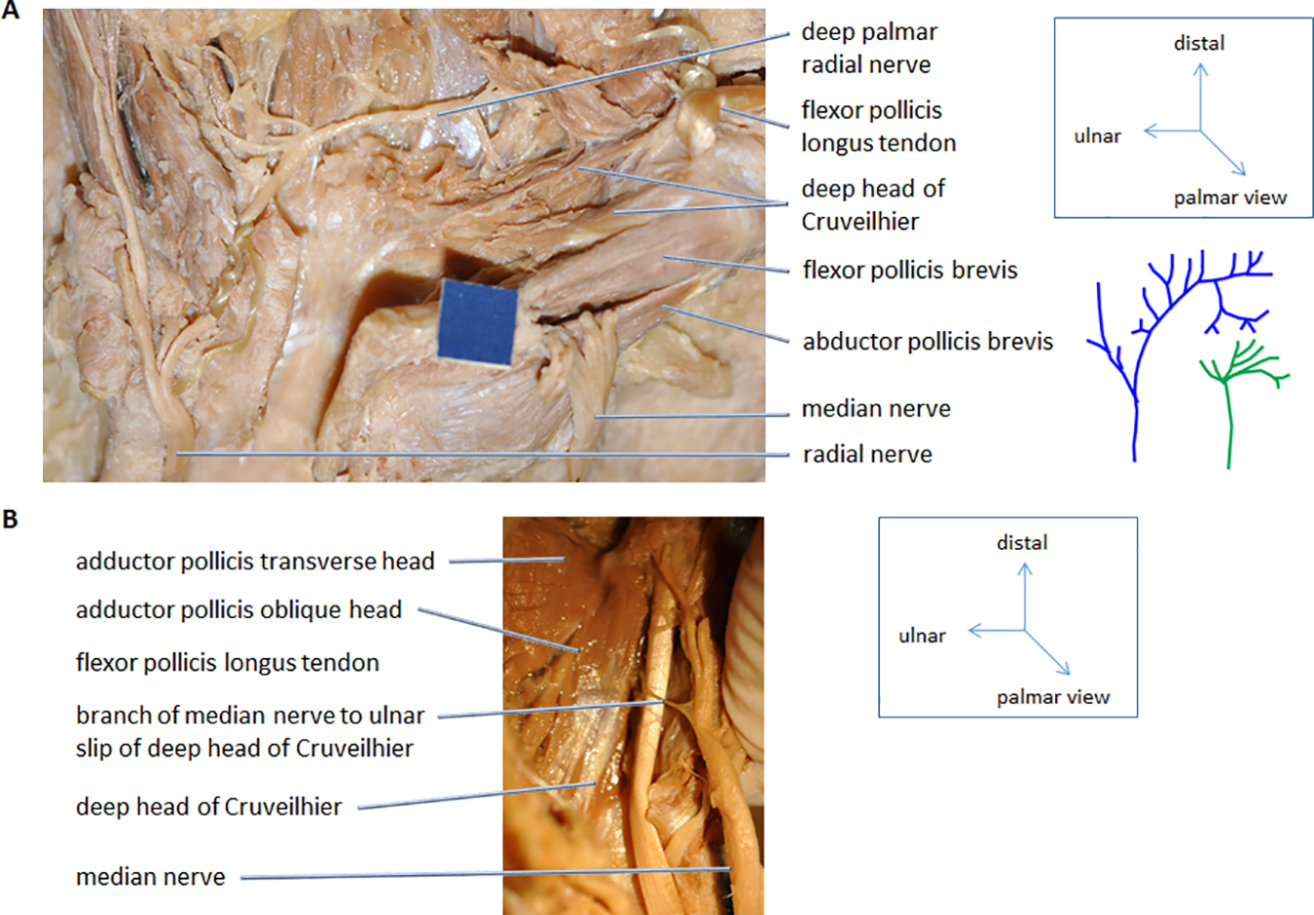
Innervation of the deep head of Cruveilhier. Palmar view of right hands. **A)** The dissection of the radial and median nerve (#766R). After dissection the schematic drawing on the right were done for all hands. **B)** The median nerve mostly innervates the radial head of the deep head of Cruveilhier. In cases where the median nerve is also innervating the ulnar head the branch usually passes under the tendon of the flexor pollicis longus. The exception is shown here (#779R). Blue scale = 1 cm.

**Fig 5:**
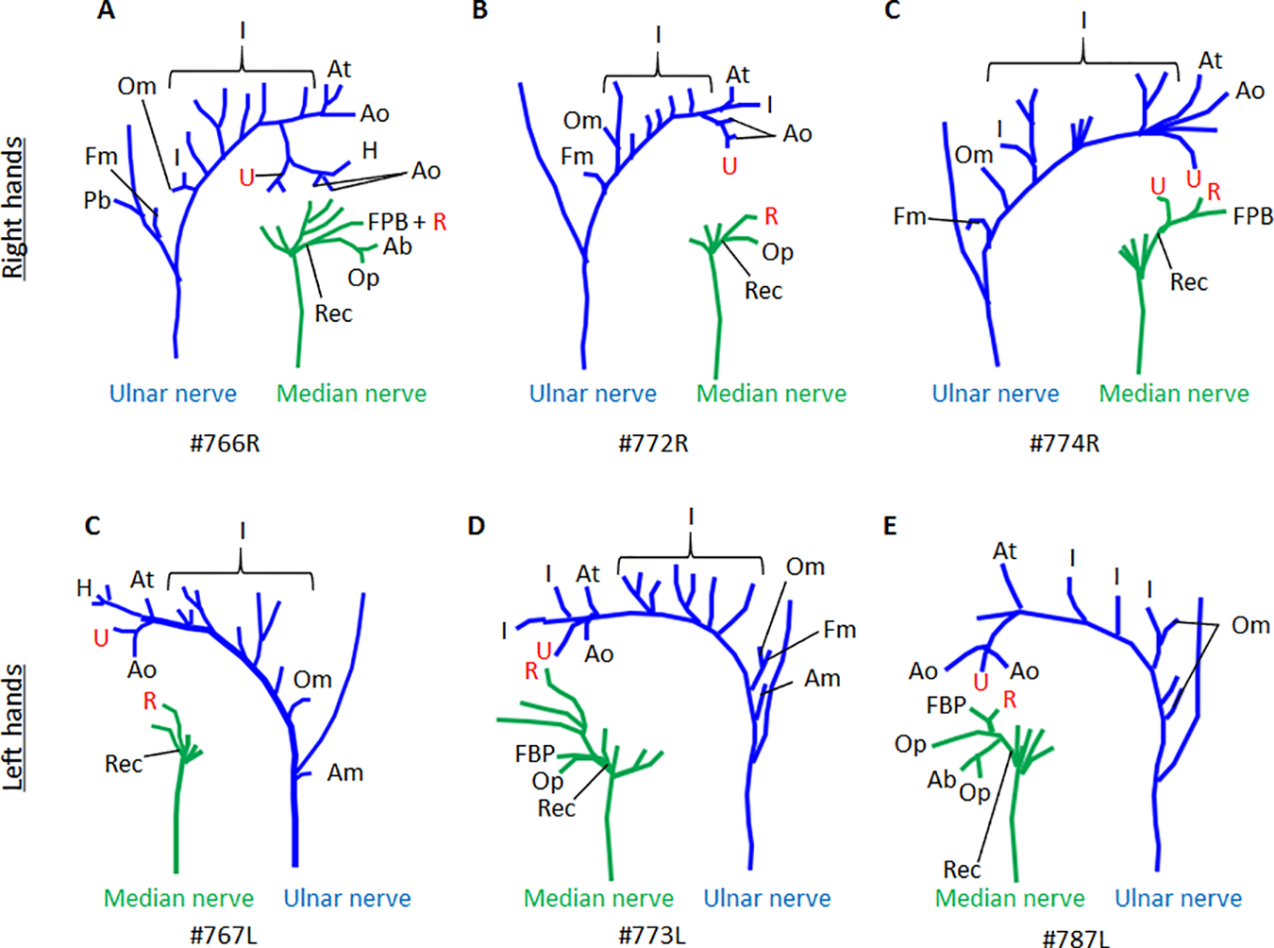
**Palmar view of dissected ulnar and median nerve in hands indicated in A-E.** Variability of branching pattern in both nerves is obvious. All hands have a split deep head of Cruveilhier with insertions onto the ulnar and radial proximal phalanx. The ulnar slip is always innervated by the ulnar nerve and the radial slip by the median nerve, except for C (#774R) where the ulnar head receives innervation from both nerves. The recurrent nerve (Rec) of the median nerve innervates the flexor pollicis brevis (FPB), the abductor pollicis brevis (Ab), and the opponens pollicis (Op). The deep palmar ulnar nerve innervates the palmar and dorsal interossei (I) while crossing the palm from medial to lateral (towards the thumb). In the thenar compartment it branches and innervates the adductor pollicis transverse (At) and oblique (Ao) heads, the muscle of Henle (H), and the first dorsal interossei (the terminal I). The innervation of the ulnar (U) and radial (R) heads of the deep head of Cruveilhier is indicated in Red to for better visualization. The palmaris brevis (Pb) is innervated by the superficial branch of the ulnar nerve and the hypothenar muscles, opponens digiti minimi (Om), flexor digiti minimi (Fm), and abductor digiti minimi (Am) are innervated by the deep ulnar nerve. One exception to this pattern is shown in Fig 5A.

The complete raw results of our study are shown in S1 Table. The results with respect to the attachments of the deep head of Cruveilhier compared with those reported by Day and Napier [34, 35] are summarized in Table 1. We also followed the ulnar and median nerves to their terminal ends in the hand and have summarized the results for the superficial head of the FPB and the deep head of Cruveilhier in Table 2. In the text, we provide a short summary of the results, which are also shown in Figs 2–5 and S1–S3 Figs. In every hand, we confirmed the correct identification of the deep head of Cruveilhier by recognizing the following: the superficial flexor pollicis brevis (FPB), the oblique head of the adductor pollicis, and the interosseous palmaris volaris (IPV) of Henle (S1 Table), with which the deep head of Cruveilhier was often confused.

**Table 1:** Day and Napier’s [34, 35] study of the deep head of Cruveilhier compared to the results of the present study.

Deep head of Cruveilhier	Day & Napier	% Day & Napier	Present study	% Present study
Absent	3	4.6	0	0
Insertion at radial sesamoid	53	81.5	35	43.75
Split insertions	8	12.4	37	46.25
Insertion at ulnar sesamoid	1	1.5	8	10
Hands in total	65	100	80	100

H0 = both populations show same distribution of slips of Cruveilhier; Χ2 = 40.77, k = 4, 3 degrees of freedom of distribution, α = 0.05 (significance level), Χ2(k-1) = 7.82; 40.77 ≥ 7.82; reject H0; Thus, the probability is less than 5% that such a large observed difference could have appeared by chance.

**Table 2:** Innervation of the heads of the flexor pollicis brevis.

Innervation	Brooks* [23]	Day and Napier [34]	Day and Napier [34] using Brooks’ method	Present study
**superficial head**				
Ulnar	5	6	2	0
Median	2	17	6	11
Ulnar & Median	19 + 5 (superficial & deep head)*	7	2	0
Total cases	31	30	10	11
**deep head of Cruveilhier**				
Ulnar	**-**	16	5	0
Median	**-**	3	3	3^a^^,^^b^
Ulnar & Median	**-**	5	2	8^a^
Total cases	0	24	10	11

*Brooks [23] inner head is not listed here because it was actually Henle’s muscle he was describing. In no hand we observed an anastomosis between the deep ulnar nerve and the median nerve.

a = ten analyzed hands were with a split deep head of Cruveilhier; 7/10: innervation of ulnar head by ulnar nerve & radial head by median nerve; 2/10: both heads innervated by median nerve; 1/10: median nerve innervates both heads and ulnar nerve innervates ulnar head

b = one hand with simple deep head of Cruveilhier inserting onto the radial sesamoid.

All superficial heads of the FPB originate from the flexor retinaculum and the trapezium (S1 Table). All but one hand (#739) had an additional origin of the superficial FPB from the wall of the carpal tunnel and one hand (#742) had an additional origin from the trapezoid. In all cases the superficial FPB inserted onto the radial side of the base of the proximal phalanx. There was a sesamoid bone in its insertion tendon. About half of the FPB (36 out of 80 hands; 45%) have additional insertions onto the distal, palmar metacarpal I shaft (12 out of 36 hands) or with the opponens pollicis (9 out of 36 hands) or with both of those locations (15 out of 36 hands).

The origin of the deep head of Cruveilhier was in all but one hand from the trapezoid, the capitate, and the ligamentum carpi radiatum (S1 Table; Fig 2). In hand #735 we found no origin from the trapezoid. This origin is almost always superficial to the oblique head of the adductor pollicis with only one exception found by us, i.e., in a single hand (#785) the deep head originated beside (proximal to the carpus) the oblique head of the adductor pollicis and was clearly separate from it. In almost all hands with a shared, continuous origin of the superficial head of FPB and the deep head of Cruveilhier, the separation of the two heads occurred within a few millimeters of the origin (Fig 2A). In eight hands, it was difficult to separate the superficial FPB and the deep head of Cruveilhier at or within a few millimeters of their origins because the heads shared a common and continuous origin from the distal flexor retinaculum, the trapezium, the distal carpal tunnel wall, and the capitate, except for one hand (#742) in which the superficial head originated additionally from the trapezoid (S1 Table). In two (#747, #755) out of those eight hands the separation between both heads could be made distally, i.e., further than a few millimeters away from the shared origin. Several deep head of Cruveilhier muscles showed additional origins (S1 Table): from the base metacarpal II (1 hand: #753), the base of metacarpal III (20 hands), the base of metacarpal III and IV (4 hands), and/or via a rough, fibrous extension of the carpal tunnel wall (43 hands).

With respect to the insertion of the deep head of Cruveilhier it is noteworthy that about half of our dissected hands (37 out of 80 hands) had two heads (Table 1, S1 Table, Figs 2A and 3), one head inserting onto the ulnar and the other onto the radial sesamoid at the proximal phalanx I. From the single deep heads of Cruveilhier (43 hands) the majority (35 hands) inserted radialward with the superficial head of the FPB onto the common tendon inserting on the radial side of the proximal phalanx I (Fig 2B, S1 Fig) and the rest (8 hands; Fig 2C, S2 Fig) ulnarward with the oblique head of the adductor pollicis. Of the eight hands with an ulnarward insertion only, four were difficult to separate from the oblique head, yet their origins were clearly recognizable by commencing from the capitate by distinct tendons lying superficial to the oblique head of adductor pollicis.

Although our percentages differ from those of Day and Napier [34] (Table 1), we encountered the same insertion sites reported by them. While the overwhelming majority of insertions in Day and Napier’s [34] study were onto the radial sesamoid we found a more or less equal distribution of split insertions and insertions onto the radial sesamoid. The deep head of Cruveilhier inserted radialward in 35 of our 80 hands (43.75%), ulnarward in eight of our 80 hands (10%), or was divided and inserted both radialward and ulnarward in 37 hands out of 80 hands (46.25%). We encountered no absences of the deep head of Cruveilhier in any of our samples. A statistical test (Chi square goodness of fit) was performed to identify any significant combinations of relationships between the scored observations (Table 1). We found that there is a significant difference in the percentage of observed insertions. That means that the differences in the number of attachments to ulnar, radial, or both sides onto the proximal phalanx in both samples is not likely (less than 5%) to have been arisen by chance.

We also dissected 11 hands to analyze the variability of innervation of the superficial FPB and the deep head of Cruveilhier (Figs 4 and 5) as was reported by Brooks [23] and Day and Napier [34] (Table 2). Brooks’ [23] inner head is not listed in our Table 2 because it was actually Henle’s muscle which he was describing. In all the hands observed the superficial FPB was innervated by the median nerve (recurrent branch). Ten of our 11 dissected hands for innervation had split deep heads of Cruveilhier, i.e., they had both ulnarward and radialward insertions. Seven of those ten hands had a median nerve innervation for the radial head and an ulnar innervation for the ulnar head; two out of ten cases were innervated by the median nerve; in one case the median nerve innervated both heads but the ulnar head, additionally, received innervation by the ulnar nerve. In the single case in which the deep head of Cruveilhier was inserting only to the radial sesamoid, it was innervated by the median nerve. In one hand (#779, Fig 4B) the nerve supplying the ulnar head originated from the median nerve and crossed the tendon of the flexor pollicis longus superficially while in the other cases the median nerve ran deep to this tendon.

Our results regarding the innervation of both superficial FPB and deep head of Cruveilhier differ from those of Brooks [23] and Day and Napier [34], respectively. Most superficial FPB were innervated by the median nerve (present study; [34]) or by both nerves [23]. The deep head of Cruveilhier was mostly innervated by the ulnar nerve according to Day and Napier [34], which would be consistent with our results if we only consider the ulnar heads of the split muscles. Comparing the results from Tables 1 and 2 with each other in respect to Day and Napier [34] the innervation differences cannot be due to an overwhelming identification of ulnar heads by Day and Napier because they identified mostly radial inserting deep heads of Cruveilhier but have a majority of ulnar innervated deep heads (see discussion).

The branching pattern of the deep palmar ulnar nerve and the median nerve vary among all dissected hand (Fig 5) as does the number of slips of thenar muscles (S3 Fig).

## Discussion

We undertook this research in order to test the assertion advanced by Day and Napier [34, 35] regarding the actual evolutionary morphology and its crucial role in the incremental improvements in thumb movements during various precision grips and handling actions. Their work on the FPB and the works of Bello‐Hellegouarch et al. [40] and Dunlap and Aziz [12] on Henle’s [26] pollical palmar interosseous has more firmly resolved the question about the actual number of thenar and neighboring area muscles in the complex actions of the thumb joints, especially the trapezio-metacarpal joint. These morphological investigations augmenting the findings of Kuhlmann and Guerin-Surville [50] and Van Sint Jan and Rooze [51] that the thumb muscles are quite variable, offering selection a rich population of muscle modifications from which to fashion the evolving complexity of thumb actions.

### Gross anatomy of the deep head of Cruveilhier

For the first time, we have empirically delineated the deep head of Cruveilhier from the neighboring muscles with which it was previously confused. In each hand we identified the deep head, the superficial FPB, the IPV of Henle, and the oblique adductor pollicis head to ensure that we not misidentify any other muscle for the deep head. Day and Napier [34, 35] did not convincingly isolate the deep head from these other muscles. However, Day and Napier [34] aimed to analyze the presence of the radial attachment of the deep head of Cruveilhier while our focus was to analyze the variability of this muscle. We have documented the same variety of the insertions of the deep head as previously shown by Day and Napier [34, 35]. However, we could not identify a hand without a deep head or without the IPV of Henle (Table 1). Our data support Day and Napier’s [34] (see also [15]) hypothesis that, based on its attachments, the deep head of FPB exists as was first proposed by the French anatomists (e.g., [52]).

The deep head is nearly always identified as originating from the capitate [8, 15–17, 34, 53–63]. Additionally, we have included as important landmarks the trapezoid and the ligamentum carpi radiatum ([59]: p103, Fig 94). The distinct and invariable pattern of this ligament (see our Fig 1A) is an invaluable aid in identifying variations of origins in both heads of FPB and the oblique head of adductor pollicis. Very similar depictions of this ligament may also be found in Zancolli and Cozzi ([64]: p19, Fig 6-3D and pp474-5, Fig 6-14A) and Ross and Lamperti ([65]: p247, Fig 1.2B). Additionally to the trapezoid, capitate, and ligamentum carpi radiatum we have registered as deep head variations of origin (S1 Table): the radialward wall of the carpal tunnel and the bases of the metacarpals II, III, and IV.

The most radialward portion of the oblique head of the adductor pollicis was regarded by Lewis [45] as contributions to the deep head of Cruveilhier. However, we regard this portion as part of the deep head because it lies over the oblique head as shown in Lewis’ figure ([45]: Fig 9.8C, p162) and as shown here, the origin of the deep head of Cruveilhier always lies superficial to the oblique adductor pollicis head. To our knowledge, this was not previously published in any study recognizing the presence of the deep head of Cruveilhier [8, 15–17, 34, 53–58, 60–63, 66–69]. However, it was shown to do so in ten illustrations [8, 16, 34, 53, 57, 59, 61, 63, 66, 68] and three studies [54, 58, 60] described its origin as blended or in common with the oblique head.

The insertion of the deep head of Cruveilhier shows variations as previously described by Day and Napier [34]: a single head can insert radialward or ulnarward, or the head can split and insert both radialward and ulnarward onto the proximal phalanx (Fig 2, Table 1). A statistical test revealed that there is a significant difference in the number of observed insertions between our study and that of Day and Napier [34]. We have a much higher percentage of split deep heads than in any other study regarding this muscle so far: 46% (present study) vs. 12.4% [34]. Most deep heads insert onto the radial sesamoid (90%; 72 out of 80 hands; including the 37 hands with a split insertion). Our different results from the previous observations in the attachments of the heads of the deep head of Cruveilhier most probably come from the special attention we paid to both the origin and insertion of this muscle and the surrounding muscles. Observed differences between our data and the one by Day and Napier [34] cannot be explained by the differences in sample size, which is 80 and 65, respectively.

Notably, variations in the details of attachments are natural as long as they are not random–i.e., as long as it is in a specific space and functional. Many studies before and after Day and Napier’s papers on the FPB have revealed striking variations of specific hand muscles: 1) IPV of Henle [12, 36, 39, 40, 50, 70]; 2) deep extensors of the forearm and hand [71]; 3) lumbricals [72, 73]; and 4) thenar muscles [50, 51, 74].

In order to analyze the effect and cause in the functional motion of the thumb versus anatomical variations (of the thumb joints, muscle slips, muscle attachments, and innervation) a good start will be a review of contemporary views relating to the origin and diversification of the precision grip, with special reference to hominid tool use and manufacture. It should be kept in mind that the power and precision grips were acquired after the morphology was in place [75]. Therefore, the observed variability supports the view that selection favored, parallel to the hominization, pollical movements. We observed in our study variability in muscles attaching to the thumb, with respect to number of slips (S3 Fig; see also [51]), innervation (e.g., Table 2), and attachments (e.g., Table 1), all characters that underlie selection. Many thenar muscles have several slips (e.g., our S3 Fig) and those variations in origins, insertions, and/or number of slips is what selection can work on. However, the observation of fascicles in hands is also dependent on the embalming process, because with increasing duration of embalming or a dissection too long after embalming might lead to the drying of the specimen, what in turn can cause the false identification of fascicles [51]. Still, the variability of pollical muscles and the form of the metacarpo-phalangeal joint enabled a range of movements advantageous for tool use; in turn the use of tools influenced the anatomy; and selection can act on variability permitting a better movement of the thumb, which aided incremental tool use facilitating tool making and finer movements of the thumb.

### Functional evidence

The actual aim of Day and Napier’s study is contained in their figure showing the varieties of insertion of Cruveilhier’s deep head ([34]: p125, their Fig 2) and illustrating how the deep head shifted its insertion from the ulnar to the radial sesamoid. Their transition series from ulnar to radial attachment also shows the migration path of the ulnar nerve into the thenar compartment. Day and Napier [34] believed that the ultimate cause of the formation of the dual-headed FPB was related to selective pressures promoting the evolution of incremental graded fine precision grip in the Old World anthropoids–especially amongst the immediate human ancestors. Day and Napier [34, 35] showed how selection acted on a variable deep head of Cruveilhier to achieve substantial migration of its insertion tendon from the ulnar metacarpo-phalangeal sesamoid to the radial one. They stated that the FPB heads flexed the trapezio-metacarpal joint of the *adducted* thumb. When the thumb was *abducted* they amplified the motive power of the metacarpal during circumduction (i.e., opposition)–movements which are crucial for various precision (fine) actions of the thumb in relation to the tips or pulps of the fingers. Day and Napier [34] proposed that this comprehensive synergy of the FPB heads was a major criterion for regarding them as a dual-headed yet singular functional (named) entity. Furthermore, they argued that the switch from the ulnarward to radial ward insertion by the radialward slip of the oblique adductor pollicis forming the deep head of the flexor pollicis brevis augmented precision grip in primates.

Subsequently, Day and Napier [35] undertook a large-scale study of various extant prosimians, monkeys (New and Old world), and of the hominoids (apes and man) to document the occurrence of the deep head of FPB in primates which showed different forms of hand anatomy and usage. This study recalled Brooks’ [23] earlier study of the thenar and hypothenar muscles of primates. Day and Napier [35] claimed that the deep head of FPB was absent in gibbons (*Hylobates*) and amongst African pongids (*Gorilla* and *Pan*) and present in Old World monkeys, *Pongo* (which they considered “anomalous” with regards to this trait), and in humans. They inferred the presence of a positive correlation between a truly opposable thumb and the presence of the FPB deep head in those primates. Day and Napier’s ([35]: p132) preposition that he diagonal obliquity of the deep FPB caused by the radialward shift of its insertion amplified “… the movements of flexion and medial rotation at the carpo-metacarpal joint; and these are the displacements and rotations which, together with abduction, compositely constitute the movement of opposition of the thumb …” has been convincingly contested by Lewis [45]. Lewis [45] reasoned that the recruitment of a slip of the adductor pollicis (acquired from the contrahentes) to bolster the FPB superficial head was “no unique innovation.” Also, according to him the oblique orientation of the deep head was a visual misperception due to its two-dimensional graphic representation in the flat diagram; in the naturally abducted thumb this “obliquity” disappears. Thus, Day and Napier’s [35] contention that the FPB deep head is a muscular novelty recruited from the adductor muscle mass to amplify the precision activities of the thumb is conjectural, at best.

According to Lewis ([45]: p165) the form of the deep head was shaped by the orientation and attachment of the middle part of the opponens pollicis onto the metacarpal; the deepest slips of this portion of the opponens “… occupying the original unexpanded origin, …” constituted the “deep head”. Although Lewis [45] makes a very convincing case (see below) to derive the FPB heads from the 2nd flexor brevis profundus (of the thumb) he does not discuss its variable insertions as shown by Day and Napier [34]. It is still quite possible that the deep head, a derivative of the flexores breves profundus, has shifted its insertion from a range including the ulnar sesamoid to the radial one under selective pressure favoring a stronger, more efficient and variable precision grip [76, 77].

Day and Napier [35] also argued that the deep carpal tunnel/arch which accommodates the thick tendons of both extrinsic finger flexors was the cause of the osteology-based specific type of thumb opposition of these African pongids (*Gorilla* and *Pan*). Those osteo-arthrological specializations of the wrist area of these taxa–i.e., the “in-set trapezium” and “in-set thumb” of these “structural brachiators”–were proposed as the cause of the diminution/absence of the deep FPB in them. Lewis [45] refuted Day and Napier’s [35] claim that the deep head of FPB was absent in *Pan*, *Gorilla*, and *Hylobates* due to the “in-set orientation” of the trapezium related to the augmentation of the depth of the carpal tunnel/arch and recent anatomical studies of *Hylobates*, *Gorilla*, and *Pan* have found the deep head in all these genera [78–80]; thus the presence of the deep head in *Pongo* was not as “anomalous” as claimed by Day and Napier [35].

### Ontogeny and evolution

Day and Napier’s [35] preliminary historical evaluation of the adaptive radiation of the hand muscles in various vertebrate taxa led them to hypothesize that the deep head of FPB was derived from contrahentes (adductor) layer of the mammalian intrinsic palmar musculature [23, 33, 81] from which the adductor pollicis of human has also been derived ([34]: p128) while its superficial head arose from flexor brevis superficialis layer. However, none of the studies supporting this origin of the deep head [23–25, 33, 81–83] investigated the variation of the muscle itself to support their hypothesis that a slip of the adductor (contrahentes) complex could actually shift its insertion radialward to form the dual-headed (“composite”) FPB. Cunningham [82] argued that this shift illustrated the hypertrophy of the adductor (contrahens) pollicis at the expense of the ulnar head of the FPB. Brooks [22, 23] instead, interpreted this shift as mechanism by which branches of the deep ulnar nerve encroached into the domain of the median nerve causing muscles via ulnar-innervated muscle slips to shift their insertion radial ward, thereby extending the domain of the ulnar nerve into the territory of the median nerve. He called these muscles “bridges” and proposed that this was the mechanism by which some thenar muscles became dual innervated. However, persuasive doubts have arisen regarding the derivation of the deep FPB from the adductor pollicis (i.e., contrahentes) complex, in studies of the development of the human hand.

Cihak [41] correlated the ontogeny and phylogeny of the intrinsic muscles of the hand and foot (see also [42]). He found that only the thenar muscles deviated from the known comparative morphogenesis of the palmar musculature. However, he noted one special exception: “Only the deep head of the flexor pollicis brevis as a part of the flexores breves profundi layer and the adductor pollicis as derived from the contrahentes layer can be set into the scheme of palmar musculature without difficulties.” ([41]: p126). Therefore, Cihak’s [41] study does not support Day and Napier’s contention that the FPB deep head has been derived from the contrahentes/adductor layer [35] or from the FPB [34] of intrinsic manual muscles. Instead, Cihak [41] proposed that (1) the abductor pollicis brevis (median nerve supplied) differentiated from the flexores breves superficialis and (2) the remaining thenar compartment muscles (median and ulnar nerve supplied) were the derivatives of a deep-level blastema ([41]: p135, Fig 104). The latter blastema belongs to the flexores breves profundi layer and is the source of the opponens pollicis and the two FPB heads and Cihak [41] showed that both heads of the FPB differentiate from the deep blastema located near metacarpal 1. The opponens pollicis derives from the flexor brevis profundus 1 and the IPV of Henle, part of the first dorsal interosseous and the FPB derive from the flexor brevis profundus 2. Cihak ([41]: p134) emphasized that the blastema complex which gave origin to both FPB heads “… does not include the adductor pollicis, which is derived from the contrahentes layer, …” However, he conceded that myoblasts from the contrahentes layer were also, most likely, involved in the formation of the interossei.

Cihak [41] found that the more radial ward derivates of the deep blastema located in the immediate vicinity of the pollical metacarpal, the opponens pollicis and the superficial head of the FPB, received the median nerve and the more ulnarward part, which formed the deep head of FPB, received branches of the deep ulnar nerve. In his illustration ([41]: p135, Fig 104) he also shows that the median nerve pierces the abductor pollicis brevis, the opponens pollicis and the FPB superficial head, whereas the deep ulnar nerve supplies the FPB deep head, the adductor pollicis complex, and the first dorsal interosseous. The important point to notice is that the deep blastema is pierced by some of the terminal branches of the (recurrent) median and the deep ulnar nerves.

### Phylogeny

In order to examine the origin of the deep head of Cruveilhier several studies turned to phylogenetic analysis and research by Lewis [37, 45], Diogo and Abdala [84], and Diogo and Wood [85, 86] have found convincing facts (including the reinterpretation of earlier works) which argue against the Cunningham-Brooks-Day and Napier’s derivation of the deep FPB “… from the contrahentes layer of the mammalian palmar muscles by radial migration, …” ([35]: p132). According to these phylogenetic investigations and the ontogenetic (and phylogenetic) research by Cihak [41, 42] the FPB heads are derived from the dual-headed flexores breves profundi layer of the mammalian palmar muscles (NB: Incidentally, this view is closer to Cunningham's [28] original conceptual scheme regarding the derivation of the pollical flexor.) Cunningham [28, 29] was, to our knowledge, the first to suggest that the flexores brevis profundi are the true progenitors of the entire FPB muscle. Day and Napier [34] first assumed that the deep head of Cruveilhier is part of the superficial FPB based on their morphological, functional, and phylogenetic analysis and later contradicted themselves stating that the deep head is actually part of the adductor pollicis [35].

Eutherian mammals like the rat have ten flexores brevis profundi in the paws of the forelimb, which insert onto the radial and ulnar sides of the five digits [45]. Lewis [45] proposed that the opponens pollicis was derived from the first flexor brevis profundus and the whole FPB in primates derived from the flexor brevis profundus 2 of the primitive paired flexors to each digit. However, Lewis ([45]: p165) did concede that there was a “germ of truth in this belief” that *occasionally*, slip(s) from the neighboring adductor pollicis oblique head got incorporated into the FPB’s deep head, which would be in accordance with McMurrich’s [33] idea of an entirely compound FPB. That is, according to McMurrich the FPB heads derived (phylogenetically) from different layers of intrinsic hand muscles. The median nerve innervated superficial head was proposed to derive from the flexores brevis superficialis and the ulnar innervated deep head of the FPB and oblique head of the adductor pollicis from the contrahentes/adductor layer.

Lewis [45] reviewed Day and Napier’s [35] contention that the FPB deep head was a slip of the oblique adductor pollicis which had repositioned its insertion from the ulnar sesamoid to the radial one and Diogo and Abdala [84], Diogo and Wood [85], and Diogo et al. [80] have revised the derivation of specific hand muscles from their ancestral intrinsic layers based on newer and expanded observations on various mammalian orders. Diogo and colleagues refined and augmented Lewis’ [45] formulation as follows: The flexores breves profundi are the true antecedents of both FPB heads [86]. Specifically, they argue that flexor brevis profundus 1 differentiates into the FPB superficial head (contrary to [41]) and the opponens pollicis (both commonly supplied by the median nerve) and the flexor brevis profundus 2 forms the deep head of the FPB (supplied by the ulnar nerve). Based on those studies, the derivation of the deep head of FBP from the oblique head of the adductor pollicis, i.e., from the contrahentes/adductor layer, cannot be sustained.

Diogo and Wood [85, 86] and Diogo et al. [13] contradict Day and Napier’s [35] and Dunlap and Aziz’s [44] earlier suggestion that the deep head of FPB is absent or cannot be observed in apes, i.e., in *Hylobates*, *Pan*, and *Gorilla*. Day and Napier [35] observed the FPB deep head in *Pongo*. This view is contrary to the hypothesis by Susman [87] that the deep head of FPB is derived in modern humans—this muscle is in fact present in most primates [86]. Also, contrary to Day and Napier [35], the FPB deep head occurs in the anthropoids (Anthropoidea, syn. Simiiformes: New World and Old World monkeys, and apes including humans). In most primates the flexor brevis profundus 2, which inserts to the ulnar side of the proximal phalanx of the first digit, is usually called the deep head of the flexor pollicis brevis [86]. The flexor brevis profundus 2 is usually present as distinct muscle in Strepsirrhini (syn. Strepsirhini; *Lemur*, *Propithecus*, *Loris*, *Nycticebus)* and Tarsiiformes (*Tarsius*, *Carlito)*, but in these taxa the muscle is often labelled as “the oblique head of the adductor pollicis” or as “the deep head of flexor pollicis brevis” [86]. The muscle is furthermore described as present in Catarrhinae, which include the Old Word monkeys (Cercopithecoidea) and Hominoidea. The deep head was described in *Papio* [23, 35, 86], *Macaca* [34, 35, 86], *Cercopithecus* [35, 45], variably present in *Colobus* [23, 43, 86], *Hylobates* [83, 86], and *Pongo*, *Pan*, and *Gorilla* [86]. However, the New World monkeys (Platyrrhini: *Aotus*, *Callithix*, *Pithecia*, and *Saimiri*), *Tupaia* (Scandentia), and *Cynocephalus* (Dermoptera), do not possess a deep head of the FPB [22, 23, 35, 43, 86]. The distribution of the presence/absence of the flexor brevis profundus 2 indicates that the muscle is present in the last common ancestor of primates and was lost or fused with its immediate neighbors in New World monkeys [86].

Analyzing in detail the photos in Diogo and Wood’s book [86] and the references mentioned above and by Diogo and Wood [86] led us to conclude that Strepsirrhini, Catarrhinae, and Tarsiiformes, have two heads of the FPB with the superficial head attaching radialward and the deep head ulnarwards on the thumb; Platyrrhini have only one superficial FPB inserting radialward; and Catarrhinae have two inserting heads. An insertion of the deep head onto both the radial and ulnar side of the proximal phalanx was reported for *Macaca*, *Semniopithecus*, and Humans [86].

### Nerve supply

Even though Fürbinger’s [88] nerve-muscle hypothesis has been refuted [81, 89, 90] innervation may provide useful clues to a muscle’s embryological and phylogenetic origins. Of course, nerve supply by itself cannot be used to make rigid statements regarding the ontogenetic or the phylogenetic relationships of muscles; rather it is best used amongst several criteria to establish muscle homology. Brooks [23] was the first to propose a mechanism by which the ulnar nerve gained access to the “territory” of the median nerve. He suggested that the radially-oriented muscle slip from the (oblique head) adductor pollicis migrated outwardly to the thenar sector bringing its nerve, a branch of the deep ulnar nerve, with it. He proposed that, from time to time, nerves used such muscular “bridges” to “extend” their domain (see also [82]).

It is abundantly clear that the FPB has two heads which in most cases receive dual innervation: from the recurrent branch of the median nerve and the branches of the deep ulnar nerve (Table 2; [22, 23, 34]). Day and Napier [34, 35] found that variations in the nerve supply of the superficial head (most commonly from the median nerve) and that of the deep head (most commonly from the deep ulnar nerve) did not preclude their classification as a single anatomical and functional unit. Nevertheless, the dual nerve supply of the FPB was one (among several) feature(s) that led them to accept the view that the two muscle heads were probably derived from different ontogenetic and phylogenetic sources. The nerves usually innervate the heads on their respective side of the flexor pollicis longus tendon (as also indicated in our Table 2). When the insertion of the deep head is radialward to this tendon then the deep head is mainly innervated by the median nerve; if it is ulnarward the deep head is mainly innervated by the ulnar nerve. We found only few exceptions to this pattern where the median nerve invades the territory of the ulnar nerve (Table 2; see below). This supports the data of Day and Napier [34], even if we have not found an ulnar innervation of the superficial FBP and the radial head of the deep head of Cruveilhier. This difference is likely caused by the number of hands we dissected–we might simply have not chosen a hand where the ulnar nerve supplies those muscles. Our differences in the observed innervations could also be due to the fixed material we used as compared to fresh specimens (e.g., [91]), what we will analyze in a future project.

However, this does not explain all the differences shown in Table 2. All (present study) or most [34] of the superficial FPB were innervated by the median nerve. Brooks [23] described a double innervation by the ulnar and median nerve but included in his description of the superficial FPB also the deep head. In this case, no real difference between our and Day and Napier’s [34] study is found although they describe a few ulnar or double innervated superficial heads. Concerning the deep head of Cruveilhier, Day and Napier [34] found that it was mostly innervated by the ulnar nerve, which would be consistent with our results if we only consider the ulnar heads of the split muscles (8 out of 11 hands). Our initial thought was that Day and Napier [34] may not have identified the heads properly, but the most common form in which the deep head of Cruveilhier appears in their study is as single head inserting to the radial side of the proximal phalanx (53 out of their 65 hands = 81.5%). However, following our observations the most common innervation for this radial head should be by the median nerve and not by the ulnar nerve. Also, Day and Napier [34] studied nearly 30 specimens to document the nerve supply of the FPB heads (Table 2). They concluded that variation of nerve supply was a common condition even though in most cases the superficial head received the median nerve while the deep head received the ulnar nerve, respectively. Considering, the developmental origin of the heads of FPB from the flexor brevis profundus 1 (superficial head) and flexor brevis profundus 2 (deep head) an innervation by two different nerves of each head might not come surprisingly. Following, our observations we agree with the small change, that the ulnar head of the deep head is mostly innervated by the ulnar nerve, while the radial head is mostly innervated by the median nerve. However, a careful analysis of fresh material should be undertaken to verify the innervation of all thenar muscles.

Brooks [23] suggested that a slip of the oblique head of the adductor pollicis, which fused with the FPB, may have acted as a bridge extending the domain of the ulnar into that of the median nerve. Flemming’s [24] slip B (radial head of deep FPB) is present in 6 out of 8 cases studied by him and was innervated by the deep ulnar nerve; he did not analyze the innervation of C (ulnar head of deep head FPB) because the more radial portion is already ulnar innervated. Haines [81] and Straus Jr. [90] proposed that during myogenesis muscles tend to pick up nerve twigs in their immediate vicinity. This mechanism may also be the basis of the dual nerve supply of the FPB heads. Day and Napier’s [34] transition series from ulnar to radial attachment also shows the migration path of the ulnar nerve into the thenar compartment. Cihak [41] shows how branches of the median and ulnar nerves could project into each other’s domain in individuals of a variable population of humans. We also see the true basis of Day and Napier’s ([34]: p126; see also Brooks [23]) observation: “…, while variation of the nerve supply is common, there is a tendency for the superficial head to be supplied by the median nerve (twenty-for out of thirty dissections) and for the deep head to be supplied by the ulnar…” Cihak’s [41] findings do not necessarily negate the other possibilities by which the FPB heads could have acquired dual innervation. Lewis [45] has conceded that, *occasionally*, cell derivatives of the adductor/contrahentes *may* fuse with the FPB deep head, creating a path for the ulnar to reach the territory of the median nerve.

Another problem involves the way the anatomy of the nerves is conceptualized to identify who is “invading” what. If the total FPB deep head is considered to be inserting ulnarward, as it would be for the flexor brevis profundus 2, then the median innervation of this ulnar-deep head could be considered as “invading” the ulnar territory, as the majority of muscles and muscle slips on the ulnar side of the flexor pollicis longus tendon are ulnar nerve innervated. With the radial head of some split FPB deep heads or of those deep heads with only radial insertion being on the radial side of the flexor pollicis longus tendon, and therefore on the side where the muscles are usually innervated by the median nerve, the radial muscle is “invading” the territory of the median nerve. It was previously suggested that muscles can pick up the innervation via nerves which are positioned close to them. If now the radial muscle / muscle slip takes the ulnar innervation across the imaginary line made by the tendon of the flexor pollicis longus than the ulnar nerve would “invade” median nerve territory.

Whatever the mechanism by which the FPB heads acquired their dual innervation, it created a muscle of exceptional versatility. Day and Napier [34] provide the most reliable actual observations of the innervation of the FPB heads; all previous descriptions of this subject are suspect, though suggestive [23, 82]. All studies prior to Day and Napier [34] regarding the true morphology of the FPB (including nerve supply) were concerned with elucidating its composition, actions, phylogeny, and clinical application and considered the IPV of Henle to be the FPB deep head (Table 2; [12, 40]). More recent careful observations, including micro-dissection under the microscope, show that the muscle does indeed have dual innervation as follows: the superficial head receives the median nerve and the deep one receives a branch of the deep ulnar nerve in most observed cases [91–96]. We have to add here, that the head that inserts ulnarward is mostly innervated by the ulnar nerve, while the head inserting radialward with the superficial FPB is mostly innervated by the median nerve–but this has to be studied in greater detail. In rare cases both heads receive median nerve branches only (the "all median hand", see [97, 98]) or the deep ulnar nerve branches only (the "all ulnar hand", see [97–100]). Of course there are cases in which both heads have dual or exclusive supply from the median or ulnar nerve, respectively [96, 101].

Also in primates, the superficial head is often described as innervated by the median nerve and the deep head as innervated by the ulnar head (see [86]). However, the variability of innervation can also be observed in primates. For example, Schultz [102] describes for *Tarsius bancanus* that the single FPB is innervated by both the median and ulnar nerves, while the related figure shows the radialward insertion onto the proximal phalanx of the thumb. In *Macaca* the innervation of the deep head FPB is usually via the ulnar nerve [103] but in some specimens via the median nerve [104]. For *Hylobates’* deep head of FPB it was reported to be innervated by the median nerve [83, 105]. In *Pan* the innervation is dependent on the insertion [86] similar to the observation in our study. There is no doubt that we need further detailed studies on larger samples of various non-human primates to establish the state of factual knowledge regarding the nerve supply of the muscles.

The prehensile hominid upper limb, the hand in particular, is the main appendage of exploration of the (new) environment. It is frequently exposed to injury. Early hominid tool use and manufacture exposed the forearm, wrist and (especially) the hand to all forms of risks (wounds, infections; torn ligaments; bone fractures) which would attenuate gripping movements. The selection of a versatile, dual-innervated muscle like the FPB insured survival. Without survival, the origin and diversification of the hominid precision grip is inconceivable. Therefore, a careful analysis of fresh material should be undertaken in order to verify the innervation of all thenar muscles, to analyze the innervation variability, and to specifically look for anastomosis of nerves that might indicate fiber exchange leading to any back-up mechanism to ensure functionality of the thumb.

### Maintaining variability

The variable use of the precision grip influenced the joints of the thumb but also other joints of the hand. Compared to the other digits, the thumb has a unique joint architecture. This is in particular evident in the saddle-shaped trapezio-metacarpal and the metacarpo-phalangeal joint, which enable an increased mobility of its distal segment. The trapezio-metacarpal joint is flattened in the ulnar aspect, which enables the opposition of the third, fourth, and fifth finger to the thumb [75]. A close look at the form of the metacarpo-phalangeal joint of the thumb showed that it gives reliable information about the ability to perform various precision grasps and ultimately to make tools (e.g., [87, 106]). The power and precision grips were established after the basic morphology was in place [75], but the evolution of the different grips also influenced in turn the morphology. The diversification of the precision grip causes and effects tool use (e.g., type writer, cellphone, stitching, cooking, farming, etc.) and through this the variations of the thumb (joints, muscle slips, muscle attachments, and innervation). Therefore, the observed variability is caused by the constant changing demands on our thumb. Selection acted on the myological variation of the deep thenar muscles which enabled more refined and variable opposition.

## Concluding remarks

On the morphology (i.e., attachments, delineation from neighboring muscles, and nerve supply) our findings substantially concur with those reported by Day and Napier [34]. However, we differ in our conclusions with respect to the developmental and evolutionary origin of the heads of FPB. It is possible that the developing deep head of the FPB may have acquired its deep ulnar branches from the neighboring oblique adductor pollicis. However, the fact that the deep head is most commonly supplied by the branches of the deep ulnar nerve does not necessarily mean that it is derived from the contrahentes/adductor layer of “… mammalian palmar muscles by radial migration …”[35].

Cihak’s [41] comparative embryological investigations show that the deep head of Cruveilhier is derived from the common blastema of the thumb’s flexor brevis profundus (see [41]: Fig 104, p135). Furthermore, Lewis’ [37, 45] and Diogo and Wood’s [86] analyses regarding the evolutionary myology of the mammalian intrinsic muscles also demonstrate that, contrary to earlier assertions [23, 28, 31, 33], the deep head of the FPB is not derived from the contrahentes layer, but both heads of the FPB are derived from the flexores breves profundi complex. Their common nerve supply (i.e., median nerve for the superficial head; deep ulnar nerve for the deep head) is due to the close proximity of the respective locations of the heads to those nerves during early embryogenesis [81, 89].

When Day and Napier [34, 35] wrote their papers, the actions of the thenar muscles were deduced by rather crude mechanical methods and in their descriptions of the FPB actions they do not explicitly describe their method of deducing muscle function. Yet, they sought to relate the evolution of the deep head with the evolutionarily sophisticated thumb opposition in hominids. They hypothesized that the deep head was recruited from the adductor (contrahentes) complex to provide motive power during the medial (pronation) partial circumduction of the trapezio-metacarpal articulation. Furthermore, Day and Napier [34, 35] proposed that this muscle slip from the oblique adductor pollicis migrated from the ulnar to the radial sesamoid (i.e., shifted its insertion radialward) in conjunction with the origin and development of true thumb opposition which is the anatomical and functional basis of the human precision grip.

According to Lewis [45] the deep head of the flexor pollicis brevis is not an “innovation”. Similarly, recent detailed studies of the comparative anatomy of the gibbon and siamangs, the gorilla, and the chimpanzee [78–80] show that these primates have a deep head of FPB. This contradicts Day and Napier’s [35] proposition that these apes lacked a true deep head on account of their lack of true pulp-to-pulp precision grip. (NB: Day and Napier [34, 35] recorded the presence of the deep head in the orangutan). Nevertheless, Day and Napier [34, 35] aim to relate the double-headed and double-innervated FPB as initially described by Cruveilhier [107] to the origin and elaboration of the precision grip was prescient. We agree with their proposition that the FPB heads have played a significant role in all those aggregate motions (flexion; medial rotation; abduction) which constitute the circumduction of the trapezio-metacarpal joint (i.e., “true opposability”) without which precision (and even power) handling is not possible. However, we do not agree that this necessarily required the recruitment of a slip from the contrahentes/adductor to create a novel, obliquely-directed muscle slip to form the deep head of Cruveilhier of the short pollical flexor.

Contrary to Cunningham, Brooks, McMurrich, and Day and Napier, there is no evidence to show that the FPB is necessarily a “compound” entity. We agree with Cihak [41], Lewis [45], and Diogo and Wood [86] that the versatile FPB—including its deep head of Cruveilhier—is derived from the flexores breves profundus group of intrinsic hand muscles. However, it is quite possible that in some individuals during the process of early embryological development the deep head picks up muscle slips from the neighboring oblique adductor pollicis which is derived from the contrahentes.

## Supporting information

S1 Fig Palmar view of left hand (#745). A large head of Cruveilhier with two slips inserts only onto the radial proximal phalanx. Almost all thumb muscles have several slips in this hand (see also S3 Fig). Blue scale = 1 cm.
(TIF)

S2 Fig Palmar view of left hand (#737). A single large head of Cruveilhier inserts onto the ulnar side of the proximal phalanx I. Blue scale = 1 cm.
(TIF)

S3 Fig Palmar view of left hand (#745) indicating slips of almost all thumb muscles. The numerous slips of the thenar muscles are structures selection can act on. Blue scale = 1 cm.
(TIF)

S1 Table Raw data collection of the 80 hands studied. The origins and insertions of the heads of the flexor pollicis brevis (FPB) are detailed here. The presence of the muscle of Henle is indicated (Henle). Often the deep head of Cruveilhier has two heads. The split is usually proximal (i.e., close to the origin), but in few cases it was distal. The yellow highlighted ID’s indicate the hands where the innervation was studied.
**X1** = All superficial heads of the FPB originate from the flexor retinaculum and the trapezium. Additional origin is indicated by “x” in the column below. All but one hand (#739) had an additional origin from wall of the carpal tunnel and one hand (#742) had an additional origin from the trapezoid.
**X2** = All superficial heads of the FPB insert onto the radial side of the proximal phalanx. Additional insertions are indicated by “x” in the columns below.
**X3** = In all but one hand the deep head of Cruveilhier originates from the ligamentum carpi radiatum, the capitate and trapezoid, and superficial to the origin of the oblique head of the adductor pollicis brevis (Hand #735 no origin from trapezoid). Additional origins are indicated by “x” in the columns below.
**A** = small, separate, additional muscle and tendon.
**B** = very separate from superficial head and difficult to separate from oblique head of adductor pollicis brevis.
**C** = two heads but both insert onto ulnar side of proximal phalanx I.
**D** = very small ulnar head.
**E** = opponens & superficial head very difficult to separate.
**Abbreviations:** proximal = heads separate close to origin; f–female; Henle–interosseus palmaris I of Henle; L–left; late = heads separate away from origin; m–male; metac.–metacarpal; N–nerve dissection; p–paired, R–right, radial–only radial insertion onto proximal phalanx I; s–single; ulnar–only ulnar insertion onto proximal phalanx I; x(split)–muscle has two heads inserting both ulnar and radial onto proximal phalanx I.
(DOCX)

## References

[1] Duchenne G-B. Physiologie des Mouvements. Paris: J.B. Baillière; 1867.

[2] Bunnell S. Opposition of the thumb. J Bone Joint Surg Am. 1938;20(2): 269–84.

[3] Haines RW. The mechanism of rotation at the first carpo-metacarpal joint. J Anat. 1944;78(Pt 1–2): 44–6. 17104939 PMC1272573

[4] Kaplan EB. Functional & Surgical Anatomy of the Hand 2 ed. Philadelphia: J. P. Lippincott, Co.; 1965.

[5] Kaplan EB. The participation of the metacarpophalangeal joint of the thumb in the act of opposition. Bull Hosp Jt Dis. 1966;27(1): 39–45. 5916091

[6] Tubiana R, Valentin P. Opposition of the thumb. Surg Clin North Am. 1968;48(5): 967–77. 5683794 10.1016/s0039-6109(16)38629-7

[7] Fahrer M. Interdependent and independent actions of the fingers In: Tubiana R, editor. The Hand. Philadelphia: W.B. Saunders Co.; 1981 p. 399–403.

[8] Tubiana R. The hand. Philadelphia, Penn: W.B. Saunders Co.; 1981.

[9] Valentin P. Extrinsic muscles of the hand and wrist: An introduction In: Tubiana R, editor. The Hand. 1. Philadelphia: W.B. Saunders Co.; 1981 p. 237–43.

[10] Valentin P. The interossei and the lumbricals In: Tubiana R, editor. The Hand. 1. Philadelphia: W.B. Saunders Co.; 1981 p. 244–54.

[11] Wilson FR. The Hand New York: New York: Vintage Books; 1999.

[12] Dunlap SS, Aziz MA. The interosseous palmaris I of Henle. www.drsamdunlap.com. 2013.

[13] Diogo R, Richmond BG, Wood B. Evolution and homologies of primate and modern human hand and forearm muscles, with notes on thumb movements and tool use. J Human Evol. 2012;63(1): 64–78. 22640954 10.1016/j.jhevol.2012.04.001

[14] Diogo R, Wood B. Origin, Development, and Evolution of Primate Muscles, with Notes on Human Anatomical Variations and Anomalies. Developmental Approaches to Human Evolution. 2016: 167–204.

[15] Wood Jones F. The Principles of Anatomy as Seen in the Hand With 2 Plates and 123 Text Figures. London: J. & A. Churchill; 1920.

[16] Anson BJ. Morris’ Human Anatomy, A Complete Systematic Treatise 12th ed New York: McGraw-Hill; 1966 482–4 p.

[17] Warwick R, Williams PL. Gray’s Anatomy 35th British Edition ed. Philadelphia: WB Saunders Co.; 1973.

[18] Hollinshead WH. Anatomy for Surgeons: Vol. 3 The Back and Limbs: Hoeber Medical Division, Harper &Row, New York.; 1969.

[19] Aziz MA, Dunlap SS, Ziermann JM. A Historical Review on the Muscle of Cruveilhier. Medical Research Archives. 2017;5(3): 1–26.

[20] Albinus BS, Bell A, Wandelaar J, Balfour J, Smellie W, Bell A, et al Tables of the Skeleton and Muscles of the Human Body: Balfour & Smellie; 1749.

[21] Henle J. Allgemeine Anatomie. Leipzig: Voss; 1841.

[22] Brooks HSJ. Variations in the nerve supply of the flexor brevis pollicis muscle. J Anat Physiol. 1886;20(4): 641–4. PMC1288596 17231651

[23] Brooks HSJ. On the morphology of the intrinsic muscles of the little finger, with some observations on the ulnar head of the short flexor of the thumb. J Anat Physiol. 1886;20(1): 645–61. 10.1007/bf03173410 PMC1288597 17231652

[24] Flemming W. Über den Flexor brevis pollicis und halluces des Menschen. Anat Anz II. 1887a: 68–77.

[25] Flemming W. Nachträgliche Notiz über den Flexor brevis pollicis. Anat Anz II. 1887b: 269–72.

[26] Henle J. Handbuch der systematischen Anatomie des Menschen, erster Band. Braunschweig: Vieweg und Sohn; 1855.

[27] Bischoff TLW. Beiträge zur Anatomie des *Hylobates leucisius* und zu einer vergleichenden Anatomie der Muskeln der Affen u. des Menschen. ABH Bayer akad Wiss Munchen Math Phys KL. 1870;10: 197–297.

[28] Cunningham DJ. The intrinsic muscles of the hand of the thylacine (*Thylacinus cynocephalus*), cuscus (*Phalangista maculata*), and phascogale (*Phascogale calura*). J Anat Physiol. 1878;12(Pt 3): 434. PMC1309794 17231207

[29] Cunningham DJ. Report on some points in the anatomy of the thylacine (*Thylacinus cynocephalus*), cuscus (*Phalangista maculata*), and phascogale (*Phascogale calura*), collected during the voyage of HMS Challenger in the years 1873–1876: With an account of the comparative anatomy of the intrinsic muscles and the nerves of the mammalian pes. Zool Challeng Exped 1882;16: 1–180.

[30] Cunningham DJ. The value of nerve-supply in the determination of muscular homologies and anomalies. J Anat Physiol. 1890;25(Pt 1): 31–40. 17231895 PMC1328106

[31] Young AH. Intrinsic muscles of the marsupial hand. J Anat Physiol. 1880;14(Pt 2): 149. PMC1309927 17231310

[32] Schäfer EA, Thane GD. Quain's Elements of Anatomy Arthrology, Myology, Angiology. 2 Pt2. 10 ed: Longmans Green and Co., London.; 1894.

[33] McMurrich JP. The phylogeny of the palmar musculature. Am J Anat. 1903;2(4): 463–500.

[34] Day M-H, Napier JR. The two heads of flexor pollicis brevis. J Anat. 1961;95(Pt 1): 123–30. PMC1244438 13720341

[35] Day M-H, Napier JR. The functional significance of the deep head of flexor pollicis brevis in primates. Folia Primatol. 1963;1(2): 122–34.

[36] Abramowitz I. On the existence of a palmar interosseous muscle in the thumb with particular reference to the Bantu-speaking Negro. S Afr J Sci. 1955;51: 270–6.

[37] Lewis OJ. The evolution of the mm. interossei in the primate hand. Anat Rec. 1965;153(3): 275–87. 5880319 10.1002/ar.1091530307

[38] Ikebuchi Y, Murakami T, Ohtsuka A. The interosseous and lumbrical muscles in the human hand, with special reference to the insertions of the interosseous muscles. Acta Medica Okayama. 1988;42(6): 327–34. doi: 10.18926/AMO/31005 3239437 10.18926/AMO/31005

[39] Henkel-Kopleck A, Schmidt HM. Das Ligamentum metacarpale pollicis. Topographie und funktionelle Deutung eines bisher unbekannten Faserzuges am Daumen und seine Lagebeziehungen zum M. interosseus internus. Handchir Microchir plast Chir. 2000;32: 223–30. 10.1055/s-2000-10932 11036543

[40] Bello‐Hellegouarch G, Aziz MA, Ferrero EM, Kern M, Francis N, Diogo R. "Pollical palmar interosseous muscle” (musculus adductor pollicis accessorius): Attachments, innervation, variations, phylogeny, and implications for human evolution and medicine. J Morph. 2013;274(3): 275–93. doi: 10.1002/jmor.20090 23109102 10.1002/jmor.20090

[41] Cihak R. Ontogeneiss of the skeleton and intrinsic mucles of the hman hand and foot. Adv Anat Embryol Cell Biol. 1972;46: 1–189. 5043313

[42] Cihak R. Differentiation and rejoining of muscular layers in the embryonic human hand. Birth Defects Orig Artic Ser. 1977;13: 97–110. 322751

[43] Dunlap S, Thorington R, Aziz M. Forelimb anatomy of New World monkeys: myology and the interpretation of primitive anthropoid models. Am J Phys Anthr. 1985;68(4): 499–517. 10.1002/ajpa.1330680406 3936364

[44] Dunlap SS, Aziz MA. Intrinsic hand muscles of primates with special reference to human trisomy syndromes. www.drsamdunlap.com. 2012.

[45] Lewis OJ. Functional Morphology of the Evolving Hand and Foot: Clarendon Press, Oxford; 1989.

[46] Morrison PE, Hill RV. And then there were four: Anatomical observations on the pollical palmar interosseous muscle in humans. Clin Anat. 2011;24(8): 978–83. doi: 10.1002/ca.21253 22009503 10.1002/ca.21253

[47] Lima Neto JQ, Carli AD, Nakamoto HA, Bersani G, Crepaldi BE, Rezende MRd. Prognostic factors on survival rate of fingers replantation. Acta ortopedica brasileira. 2015;23(1): 16–8. doi: 10.1590/1413-78522015230101026 26327788 10.1590/1413-78522015230101026 PMC4544513

[48] Hou J-X, Xie S-Q, Zhang H-F, Dong Q-Q, Zhang M-W, Wu Z-S, et al Replantation of hand multi-level severances with 17 segments. Injury Extra. 2014;45(6): 41–4.

[49] Romanes GJ. Cunningham's Manual of Practical Anatomy. Thirteenth Edition, Volume 1, Upper and Lower Limbs. Cunningham DJ, editor. London: Oxford University Press; 1966.

[50] Kuhlmann J, Guerin-Surville H. Agencement fasciculaire des muscles intrinsèques du pouce. Bull Assoc Anatom. 1985;69(204): 29–41. 3833310

[51] Van Sint Jan S, Rooze M. Anatomical variations of the intrinsic muscles of the thumb. Anat Rec. 1994;238(1): 131–46. doi: 10.1002/ar.1092380115 8116886 10.1002/ar.1092380115

[52] Cruveilhier J. Traité d'Anatomie Descriptive. Paris: Labe; 1843.

[53] Thomson A, Schafer EA, Thane GD. Quain's Elements of Anatomy. New York: William Wood and Co; 1882.

[54] Robinson A. Cunningham's Textbook of Anatomy. 5 ed Robinson A, editor. New York: William Wood & Co; 1923.

[55] Robinson A. Cunningham’s Text-Book of Anatomy. Robinson A, editor. New York: Oxford University Press; 1931.

[56] Hollinshead WH. Anatomy for Surgeons: Vol 3, The Back and Limbs New York: Hoeber—Harper; 1958.

[57] Wolf-Heidegger G. Atlas of Systematic Human Anatomy. New York: Hafner Pub. Co; 1962.

[58] Gardner E, Gray D, O'Rahilly R. Anatomy: A Regional Study of Human Structure Philadelphia: WB Saunders Co.; 1975.

[59] Ferner H. Pernkopf's Atlas of Topographical and Applied Human Anatomy: Thorax, Abdomen, and Extremities. Ferner H, editor. Philadelphia: WB Saunders Co.; 1964.

[60] O’Rahilly R. Basic Human Anatomy. Philadelphia: WB Saunders; 1977 566 p.

[61] Clemente CD. Anatomy of the Human Body by Henry Gray. Clemente CD, editor. Philadelphia: Lea and Febiger; 1985. 1878 p.

[62] Stern JT. Essentials of Gross Anatomy. Philadelphia: FA Davis Co; 1988.

[63] Toldt C. An Atlas of Human Anatomy for Students and Physicians 6. New York: Rebman Company; 1904 Toldt C. 1904. An Atlas of Human Anatomy, Part III. Rebman Co., New York.

[64] Zancolli EA, Cozzi EP. Atlas of Surgical Anatomy of the Hand. New York: Churchill Livingstone; 1992.

[65] Ross L, Lamperti E. Thieme Atlas of Anatomy: General Anatomy and Musculoskeletal System. Stuttgart: Thieme; 2006. 541 p.

[66] Landsmeer JMF. Atlas of Anatomy of the Hand. New York: Churchill Livingstone; 1976.

[67] Huber GC. Piersol’s Human Anatomy, Including Structure and Development and Practical Considerations. 9th Revised ed. Philadelphia: J. B. Lippincott, Co.; 1930.

[68] Woodburne RT, Burkel WE. Essentials of Human Anatomy 8 ed. New York: Oxford University Press; 1988.

[69] Schmidt HM, Lanz U. Surgical Anatomy of the Hand. Stuttgart, New York: Thieme; 2004.

[70] Witthaut J, Leclercq C. Der M. interosseus palmaris des Daumes: Ergebnisse einer anatomischen Studie zu einem Muskel des ersten Strahls. Handchir Microchir plast Chir. 1999;31(1): 66–9. 10.1055/s-1999-13496 10080066

[71] Aziz MA, Dunlap SS. The human extensor digitorum profundus muscle with comments on the evolution of the primate hand. Primates. 1986;27(3): 293–319.

[72] Basu S, Hazary S. Variations of the lumbrical muscles of the hand. Anat Rec. 1960;136(4): 501–4. 13797470 10.1002/ar.1091360409

[73] Russell K, Sunderland S. Abnormalities of the lumbrical muscles of the hand. J Anat. 1938;72(Pt 2): 306–7. PMC1252425 17104697

[74] Perkins RE, Hast MH. Common variations in muscles and tendons of the human hand. Clin Anat. 1993;6(4): 226–31.

[75] Marzke MW. Evolutionary development of the human thumb. Hand Clinics. 1992;8(1): 1–8. 1572915

[76] Marzke MW, Shackley MS. Hominid hand use in the Pliocene and Pleistocene: evidence from experimental archaeology and comparative morphology. J Human Evol. 1986;15(6): 439–60.

[77] Napier JR. The prehensile movements of the human hand. Bone Joint J. 1956;38(4): 902–13. 10.1302/0301-620X.38B4.902 13376678

[78] Diogo R, Potau JM, Pastor JF, Ferrero EM, Bello G, Barbosa M, et al Photographic and Descriptive Musculoskeletal Atlas of Gorilla: With Notes on the Attachments, Variations, Innervation, Synonymy and Weight of the Muscles: CRC Press; 2010.

[79] Diogo R, Potau JM, Pastor JF, Ferrero EM, Bello G, Barbosa M, et al Photographic and Descriptive Musculoskeletal Atlas of Gibbons and Siamangs (Hylobates): With Notes on the Attachments, Variations, Innervation, Synonymy and Weight of the Muscles: CRC Press; 2012.

[80] Diogo R, Potau JM, Pastor JF, de Paz F, Ferrero E, Bello G, et al Photographic and Descriptive Musculoskeletal Atlas of Chimpanzees (*Pan*): Oxford: Taylor & Francis; 2013.

[81] Haines RW. A consideration of the constancy of muscular nerve supply. J Anat. 1935;70(Pt 1): 33 17104574 PMC1249278

[82] Cunningham DJ. The flexor brevis pollicis and the flexor brevis hallucis in man. Anat Anz II. 1887;(10): 186–92.

[83] Brooks HSJ. On the short muscles of the pollex and hallux of the anthropoid apes, with special reference to the opponens hallucis. J Anat Physiol. 1887;22(Pt 1): 78–95. 17231730 PMC1288711

[84] Diogo R, Abdala V. Muscles of Vertebrates—Comparative Anatomy, Evolution, Homologies and Development. Enfield, New Hampshire: CRC Press; Science Publisher; 2010. 482 p.

[85] Diogo R, Wood B. Soft‐tissue anatomy of the primates: phylogenetic analyses based on the muscles of the head, neck, pectoral region and upper limb, with notes on the evolution of these muscles. J Anat. 2011;219(3): 273–359. doi: 10.1111/j.1469-7580.2011.01403.x 21689100 10.1111/j.1469-7580.2011.01403.x PMC3171772

[86] Diogo R, Wood B. Comparative Anatomy and Phylogeny of Primate Muscles and Human Evolution: CRC press; 2012.

[87] Susman RL. Fossil evidence for early hominid tool use. Science. 1994;265(5178): 1570–3. 8079169 10.1126/science.8079169

[88] Fürbinger M. Untersuchungen zur Mophologie und Systematik der Vögel. Amsterdam und Jena1888.

[89] Straus WL. Jr. The concept of nerve-muscle specifity Biol Rev. 1946;21(2): 75–91. 21023421 10.1111/j.1469-185x.1946.tb00454.x

[90] Straus WL. Jr. The pattern of the intrinsic palmar musculature. Biol Bull. 1946;91(02): 233–. 20341142

[91] Harness D, Sekeles E. The double anastomotic innervation of thenar muscles. J Anat. 1971;109(Pt 3): 461 5153804 PMC1270989

[92] Falconer D, Spinner M. Anatomic variations in the motor and sensory supply of the thumb. Clin Orthop Relat Res. 1985;195: 83–96. 3978968

[93] Homma T, Sakai T. Thenar and hypothenar muscles and their innervation by the ulnar and median nerves in the human hand. Cells Tissues Organs. 1992;145(1): 44–9. 10.1159/000147340 1414212

[94] Mumford J, Morecraft R, Blair WF. Anatomy of the thenar branch of the median nerve. J Hand Surg. 1987;12(3): 361–5. 10.1016/s0363-5023(87)80004-7 3584881

[95] Olave E, Prates J, Del Sol M, Sarmento A, Gabrielli C. Distribution patterns of the muscular branch of the median nerve in the thenar region. J Anat. 1995;186(Pt 2): 441. 7649846 PMC1167205

[96] Ajmani M. Variations in the motor nerve supply of the thenar and hypothenar muscles of the hand. J Anat. 1996;189(Pt 1): 145. PMC1167836 8771405

[97] Marinacci A. Diagnosis of "All median hand" Electromyography. 1964;4: 85 14206973

[98] Marinacci A. The problem of unusual anomalous innervation of hand muscles. The value of electrodiagnosis in its evaluation. Bull Los Angel Neuro Soc. 1964;29: 133–42. 14214210

[99] Rowntree T. Anomalous innervation of the hand muscles. J Bone Joint Surg Br. 1949;31(4): 505–10. 15397131

[100] King RB. Anomalous innervation of the hand muscles: Case report. J Neurosurg. 1951;8(5): 528–9. doi: 10.3171/jns.1951.8.5.0528 14880943 10.3171/jns.1951.8.5.0528

[101] McFarlane R. Observations on the functional anatomy of the intrinsic muscles of the thumb. J Bone Joint Surg Am. 1962;44(6): 1073–88.

[102] Schultz M. Osteology and Myology of the Upper Extremity of Tarsius. Niemitz C, editor. Stuttgart: Gustav Fischer Verlag; 1984.

[103] Kimura K, Takai S. On the musculature of the forelimb of the crab-eating monkey. Primates. 1970;11(2): 145–70.

[104] Akiyama K. The nerves of the hand of the *Macacus cyclopsis*. Okajimas Folia Anatomica Japonica. 1960;34(6): 641–55.

[105] Hepburn D. The comparative anatomy of the muscles and nerves of the superior and inferior extremities of the anthropoid apes: part I. J Anat Physiol. 1892;26(Pt 2): 149 17231981 PMC1328229

[106] Napier JR. The evolution of the hand. Sci Am. 1962;207: 56–62. 13937573 10.1038/scientificamerican1262-56

[107] Cruveilhier J. Traité d’Anatomie Descriptive. Paris: P. Assleils Liraire de la Faculte de Medecine; 1834–1836.

