# Supplementary figures and images for: Anatomical variations of the deep head of Cruveilhier of the flexor pollicis brevis and its significance for the evolution of the precision grip
Source: PLoS One. 2017 Nov 9;12(11):e0187402. doi: 10.1371/journal.pone.0187402 (PMC5679560; doi:10.1371/journal.pone.0187402)

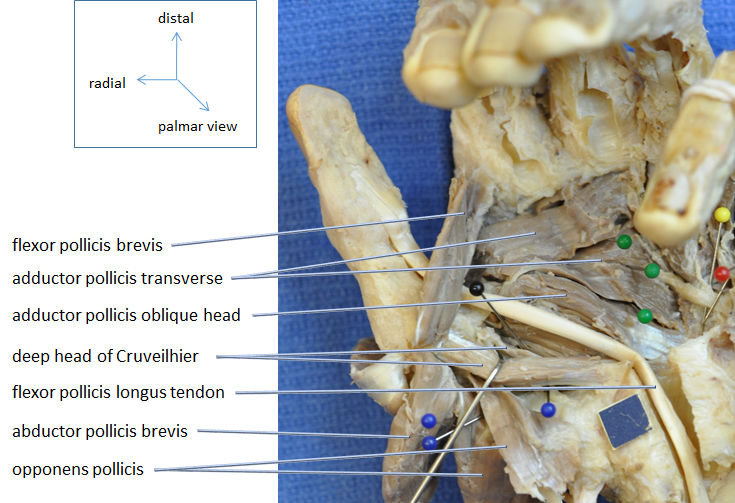

Supplement: S1 Fig — A large head of Cruveilhier with two slips inserts only onto the radial proximal phalanx. Almost all thumb muscles have several slips in this hand (see also S3 Fig). Blue scale = 1 cm. (TIF) [file pone.0187402.s001.tif]

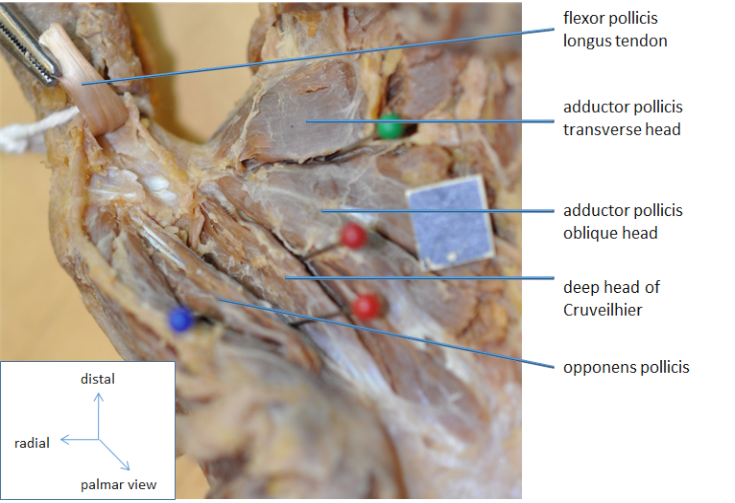

Supplement: S2 Fig — A single large head of Cruveilhier inserts onto the ulnar side of the proximal phalanx I. Blue scale = 1 cm. (TIF) [file pone.0187402.s002.tif]

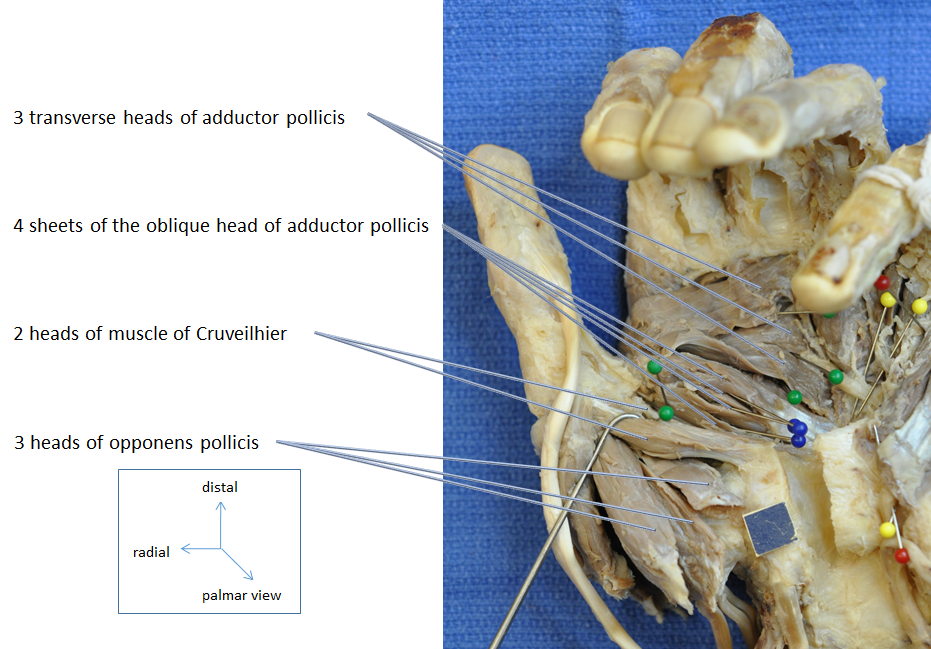

Supplement: S3 Fig — The numerous slips of the thenar muscles are structures selection can act on. Blue scale = 1 cm. (TIF) [file pone.0187402.s003.tif]
