# Supplementary material for: Anatomical variations of the deep head of Cruveilhier of the flexor pollicis brevis and its significance for the evolution of the precision grip
Source: PLoS One. 2017 Nov 9;12(11):e0187402. doi: 10.1371/journal.pone.0187402 (PMC5679560; doi:10.1371/journal.pone.0187402)
Supplement: S1 Table — The origins and insertions of the heads of the flexor pollicis brevis (FPB) are detailed here. The presence of the muscle of Henle is indicated (Henle). Often the deep head of Cruveilhier has two heads. The split is usually proximal (i.e., close to the origin), but in few cases it was distal. The yellow highlighted ID’s indicate the hands where the innervation was studied. X1 = All superficial heads of the FPB originate from the flexor retinaculum and the trapezium. Additional origin is indicated by “x” in the column below. All but one hand (#739) had an additional origin from wall of the carpal tunnel and one hand (#742) had an additional origin from the trapezoid. X2 = All superficial heads of the FPB insert onto the radial side of the proximal phalanx. Additional insertions are indicated by “x” in the columns below. X3 = In all but one hand the deep head of Cruveilhier originates from the ligamentum carpi radiatum, the capitate and trapezoid, and superficial to the origin of the oblique head of the adductor pollicis brevis (Hand #735 no origin from trapezoid). Additional origins are indicated by “x” in the columns below. A = small, separate, additional muscle and tendon. B = very separate from superficial head and difficult to separate from oblique head of adductor pollicis brevis. C = two heads but both insert onto ulnar side of proximal phalanx I. D = very small ulnar head. E = opponens & superficial head very difficult to separate. Abbreviations: proximal = heads separate close to origin; f–female; Henle–interosseus palmaris I of Henle; L–left; late = heads separate away from origin; m–male; metac.–metacarpal; N–nerve dissection; p–paired, R–right, radial–only radial insertion onto proximal phalanx I; s–single; ulnar–only ulnar insertion onto proximal phalanx I; x(split)–muscle has two heads inserting both ulnar and radial onto proximal phalanx I. (DOCX) [file pone.0187402.s004.docx]

# Supplementary Table

**S1 Table.** **Raw data collection of the 80 hands studied.** The origins and insertions of the heads of the flexor pollicis brevis (FPB) are detailed here. The presence of the muscle of Henle is indicated (Henle). Often the deep head of Cruveilhier has two heads. The split is usually proximal (i.e., close to the origin), but in few cases it was distal. The yellow highlighted ID’s indicate the hands where the innervation was studied.

**X1** = All superficial heads of the FPB originate from the flexor retinaculum and the trapezium. Additional origin is indicated by “x” in the column below. All but one hand (#739) had an additional origin from wall of the carpal tunnel and one hand (#742) had an additional origin from the trapezoid.

**X2** = All superficial heads of the FPB insert onto the radial side of the proximal phalanx. Additional insertions are indicated by “x” in the columns below.

**X3** = In all but one hand the deep head of Cruveilhier originates from the ligamentum carpi radiatum, the capitate and trapezoid, and superficial to the origin of the oblique head of the adductor pollicis brevis (Hand #735 no origin from trapezoid). Additional origins are indicated by “x” in the columns below.

**A** = small, separate, additional muscle and tendon

**B** = very separate from superficial head and difficult to separate from oblique head of adductor pollicis brevis

**C** = two heads but both insert onto ulnar side of proximal phalanx I

**D** = very small ulnar head

**E** = opponens & superficial head very difficult to separate

**Abbreviations:** proximal = heads separate close to origin; f – female; Henle – interosseus palmaris I of Henle; L – left; late = heads separate away from origin; m – male; metac. – metacarpal; N – nerve dissection; p – paired, R – right, radial – only radial insertion onto proximal phalanx I; s – single; ulnar – only ulnar insertion onto proximal phalanx I; x(split) – muscle has two heads inserting both ulnar and radial onto proximal phalanx I

|  |  | **Superficial head** | | | **Deep head of Cruveilhier** | | | |  |  |  |
| --- | --- | --- | --- | --- | --- | --- | --- | --- | --- | --- | --- |
| **ID** | **gender/paired** | **origins: X1** | **insertions: X2** | | **origins: X3** | | **insertions** | | **heads** | | **Henle** |
| R/L | f/m; p/s | carpal tunnel | distal, palmar metac. I shaft | opponens pollicis | base of metac. | ascending carpal tunnel on floor | ulnarward; prox. phalanx I | distal, palmar metac. I shaft | separated | |  |
| 700R | f,p | x | x |  |  | x | radial |  | proximal | | x |
| 701L |  | x |  |  |  | x | radial |  | proximal | | x |
| 702R | m,p | x |  |  |  | x | x (split) |  | proximal | | x |
| 703L |  | x |  |  |  |  | ulnar |  | proximal | | x |
| 704R | f,p | x |  |  |  |  | ulnar |  | proximal | | x |
| 705L |  | x | x |  |  |  | x (split) | x | proximal | | x |
| 706R | f,p | x |  |  | III | x | radial |  | proximal | | x |
| 707L |  | x |  |  |  |  | radial |  | proximal | | x |
| 712R | f,p | x |  |  | III | x | radial |  | proximal | | x |
| 713L |  | x |  |  |  |  | x (split) | x | proximal | | x |
| 716R | m,p | x | x |  |  | x | radial |  | proximal | | x |
| 717L |  | x | x | x |  | x | ulnar |  | proximal | | x |
| 718L | f,s | x |  |  |  | x | radial |  | proximal | | x |
| 719L | m,s | x |  |  |  | x | radial |  | proximal | | x |
| 722R | m,p | x |  |  |  | x | x (split) |  | proximal | | x (lig) |
| 723L |  | x |  |  |  | x | x (split) |  | proximal | | x (lig) |
| 724R | f,p | x |  |  |  | x | radial | x | proximal | | x |
| 725L |  | x |  |  | III |  | radial |  | proximal | | x |
| 726R | m,p | x | x | x |  | x | radial |  | proximal | | x |
| 727L |  | x |  |  |  |  | radial |  | proximal | | x |
| 728R | m,p | x |  |  |  |  | x (split) |  | proximal | | x |
| 729L |  | x |  |  |  |  | radial |  | proximal | | x |
| 730R | f,p | x | x |  |  | x | radial |  | proximal | | x |
| 731L |  | x | x |  | III | x | radial |  | proximal | | x |
| 732R | f,p | x |  |  | III | x | radial |  | proximal | | x |
| 733L |  | x |  |  | III | x | x (split) | x (A) | proximal | | x |
| 734R | f,p | x | x |  | III (B) |  | radial | x (A) | proximal | | x |
| 735L |  | x |  |  | III & IV (B) |  | radial |  | proximal | | x |
| 736R | m,p | x |  | x | III |  | radial | x | proximal | | x |
| 737L |  | x |  |  |  | x | ulnar |  | proximal | | x |
| 738R | m,p | x |  | x | III | x | radial |  | proximal | | x |
| 739L |  | - | x |  |  | x | x (split) | x | proximal | | x |
| 740R | f,p | x |  | x | III |  | radial |  | proximal | | x |
| 741L |  | x |  | x |  | x | x (split) |  | at origin | | x |
| 742R | m,s | x & trapezoid |  |  | III |  | x (split) |  | proximal | | x |
| 743L | m,s | x |  |  | III | x | ulnar |  | proximal | | x |
| 744R | m,p | x |  | x |  | x | radial |  | proximal | | x |
| 745L |  | x |  | x |  | x | radial |  | proximal | | x |
| 746R | f,p | x |  |  | III | x | radial |  | proximal | | x |
| 747L |  | x |  |  | III |  | x (split) | x | distal | | x |
| 748R | f,p | x |  |  |  | x | radial |  | proximal | | x |
| 749L |  | x |  |  |  | x | radial |  | proximal | | x |
| 750R | f,p | x |  |  |  |  | x (split) |  | proximal | | x |
| 751L |  | x |  |  |  |  | ulnar (C) |  | proximal | | x |
| 752L | m,s | x | x | x | III |  | radial |  | proximal | | x |
| 753L | m,s | x |  |  | II |  | ulnar | x | proximal | | x |
| 754L | m,s | x |  |  |  | x | x (split) |  | proximal | | x |
| 755L | m,s | x |  |  |  |  | x (split) |  | distal (~1cm) | | x |
| 756R | f,p | x |  |  |  | x | radial |  | proximal | | x |
| 757L |  | x | x |  |  |  | radial |  | proximal | | x |
| 758R | f,p | x | x | x | III & IV |  | radial |  | proximal | | x |
| 759L |  | x |  |  | III & IV |  | radial |  | proximal | | x |
| 760R | m,p | x |  |  | III |  | x (split) |  | difficult | | x |
| 761L |  | x |  |  | III |  | x (split) |  | proximal | | x |
| 762R | m,p | x | x | x |  |  | x (split) |  | difficult | | x |
| 763L |  | x |  |  |  |  | ulnar |  | proximal | | x |
| 764R | m,p | x | x | x |  |  | x (split) |  | difficult | | x |
| 765L |  | x | x | x |  |  | x (split) |  | proximal | | x |
| 766R | f,p,N | x | x | x |  |  | x (split) |  | proximal (B) | | x |
| 767L |  | x | x | x |  | x | x (split) |  | proximal | | x |
| 768R | f,p | x |  |  |  |  | x (split) |  | proximal | | x |
| 769L |  | x |  |  |  |  | x (split) |  | proximal | | x |
| 770R | m,s | x |  | x | III |  | radial |  | proximal | | x |
| 771R | m,s | x |  |  |  | x | x (split) |  | difficult | | x |
| 772R | f,s,N | x |  | x | III | x | x (split) | x | proximal | | x |
| 773L | f,s,N | x | x |  |  | x | x (split) |  | proximal | | x |
| 774R | m,s,N | x | x | x | III |  | x (split) | x | proximal | | x |
| 775L | m,s | x |  | x |  |  | radial |  | proximal | | x |
| 776R | m,p | x |  |  |  | x | x (split) |  | difficult | | x |
| 777L |  | x | x | x | III & IV | x | x (split) |  | difficult | | x |
| 778R | m,s,N | x | x |  |  | x | x (split) | x | proximal | | x |
| 779R | f,s,N | x | x | x |  | x | x (split) | x | proximal | | x |
| 780R | m,s | x |  |  |  |  | x (split) |  | proximal | | x |
| 781L | m,s | x | x |  |  | x | x (split) |  | proximal | | x |
| 782R | m,p | x |  |  |  | x | x (split) |  | proximal | | x |
| 783L |  | x | x |  |  | x | radial |  | proximal (B) | | x |
| 784R | f,p,N | x | x |  |  | x | x (split) (D) | x | proximal | | x |
| 785L |  | x | x | x (E) |  |  | radial | x | proximal | | x |
| 786R | f,p,N | x | x | x |  | x | x (split) | x | proximal | | x |
| 787L |  | x | x | x |  | x | x (split) | x | proximal | | x |
